# Basis of Virulence in Enterotoxin-Mediated Staphylococcal Food Poisoning

**DOI:** 10.3389/fmicb.2018.00436

**Published:** 2018-03-13

**Authors:** Emilie L. Fisher, Michael Otto, Gordon Y. C. Cheung

**Affiliations:** Pathogen Molecular Genetics Section, Laboratory of Bacteriology, National Institute of Allergy and Infectious Diseases, National Institutes of Health, Bethesda, MD, United States

**Keywords:** *Staphylococcus aureus*, superantigen, enterotoxins, food poisoning, regulation, virulence, emesis

## Abstract

The *Staphylococcus aureus* enterotoxins are a superfamily of secreted virulence factors that share structural and functional similarities and possess potent superantigenic activity causing disruptions in adaptive immunity. The enterotoxins can be separated into two groups; the classical (SEA-SEE) and the newer (SEG-SE*l*Y and counting) enterotoxin groups. Many members from both these groups contribute to the pathogenesis of several serious human diseases, including toxic shock syndrome, pneumonia, and sepsis-related infections. Additionally, many members demonstrate emetic activity and are frequently responsible for food poisoning outbreaks. Due to their robust tolerance to denaturing, the enterotoxins retain activity in food contaminated previously with *S. aureus*. The genes encoding the enterotoxins are found mostly on a variety of different mobile genetic elements. Therefore, the presence of enterotoxins can vary widely among different *S. aureus* isolates. Additionally, the enterotoxins are regulated by multiple, and often overlapping, regulatory pathways, which are influenced by environmental factors. In this review, we also will focus on the newer enterotoxins (SEG-SE*l*Y), which matter for the role of *S. aureus* as an enteropathogen, and summarize our current knowledge on their prevalence in recent food poisoning outbreaks. Finally, we will review the current literature regarding the key elements that govern the complex regulation of enterotoxins, the molecular mechanisms underlying their enterotoxigenic, superantigenic, and immunomodulatory functions, and discuss how these activities may collectively contribute to the overall manifestation of staphylococcal food poisoning.

## Introduction

*Staphylococcus aureus* is a dangerous human pathogen whose virulence potential predominantly relies on the production of an impressive catalog of protein toxins. These can work separately or in concert to cause a multitude of human diseases. Pneumonia, sepsis-related infections, toxic shock syndrome, and food poisoning are diseases that have traditionally been associated in particular with the production of enterotoxins (Lowy, 2003). However, recent studies suggest that the staphylococcal enterotoxins (SEs) have a broader role in the manifestation of a number of other human illnesses, including those associated with the respiratory tract (Pastacaldi et al., 2011; Huvenne et al., 2013) and the development of autoimmune diseases (Principato and Qian, 2014; Li et al., 2015). The SEs are powerful non-specific T-cell stimulators (superantigens) that cause unregulated activation of the immune response (for detailed reviews see Fraser and Proft, 2008; Spaulding et al., 2013). If this stimulation is sustained, a massive cytokine overload is produced preluding the clinical hallmarks of toxic shock syndrome, which is characterized by the fast onset of fever, organ failure and significant mortality (Lappin and Ferguson, 2009). Unlike the majority of other secreted toxins produced by *S. aureus*, the SEs require only minute quantities to be toxic in humans. Additionally, the SEs have a remarkable tolerance to extreme denaturing conditions, such as low pH (Schantz et al., 1965; Bergdoll, 1983), heating (Evenson et al., 1988; Asao et al., 2003) and proteolytic digestion (Humber et al., 1975; Regenthal et al., 2017). These combined qualities make the SEs, especially SEB, potential bioterrorism agents (Madsen, 2001). Notably, SEB is also classified as a Category B select agent by various United States federal agencies.

In addition to the toxic effects they have on the host, the SEs are potent emesis-inducing toxins. Reports of the involvement of enterotoxin-producing *S. aureus* in staphylococcal food poisoning (SFP) can be dated as far back as the 1900s. However, it was not until the 1930s that a link between the two were made (Dack, 1937). In healthy human individuals, SFP is an acute disease depicted by symptoms including nausea, vomiting, abdominal cramping, diarrhea, typically in the absence of fever, appearing within 3–9 h after the ingestion of food contaminated previously with enterotoxin-producing *S. aureus*. SFP is often self-limiting with recovery occurring 1–3 days after the onset of symptoms (Le Loir et al., 2003). However, symptoms may be more severe in the young, elderly and immunocompromised (Murray, 2005; Argudin et al., 2010). The SEs' ability to traverse the harsh acidic conditions within the gut to reach the intestine means that the advancement of SFP can also occur in the absence of live bacteria. Typically, only high nanogram to low microgram quantities of enterotoxins are needed to induce the symptoms of SFP (Larkin et al., 2009).

Next to *E. coli, Shigella, Bacillus* spp., and *Clostridium* spp., *S. aureus* is among the leading toxin-producing bacterial causative agents of food poisoning. *S. aureus* is also frequently mentioned in national foodborne illness estimates (Gkogka et al., 2011; Bennett et al., 2013; Thomas et al., 2013; Kirk et al., 2014; Mangen et al., 2015; Park et al., 2015; Van Cauteren et al., 2017), and is identified as a main player in major food poisoning outbreaks worldwide (Asao et al., 2003; Do Carmo et al., 2004; Chiang et al., 2008; Ostyn et al., 2010; Sato'o et al., 2014; Ercoli et al., 2017). In the US alone, it is estimated that *S. aureus* accounts for more than 240,000 foodborne illnesses per year (Scallan et al., 2011). However, considering that SFP can be resolved in individuals without hospitalization, it is not unusual for many cases to go unreported. While SFP rarely develops into a life-threatening disease, its frequency has a significant impact on the economy, resulting in a loss in productivity. It also represents a serious financial burden, especially for the food industry, catering businesses, and public healthcare systems. The implementation of traditional hygiene practices and proper food safety measures are key to preventing foodborne illness (Hussain and Dawson, 2013).

## The superfamily of staphylococcal enterotoxins; proteins and overview

The superfamily of SEs and enterotoxin-like (SE*l*s) proteins (Table 1) share many common features; they are non-glycosylated, antigenically distinct, low molecular weight (19–29 kDa) single-chain proteins that all fold into homologous globular structures (Thomas et al., 2007). Since the first characterization of the classical SEs (SEA to SEE) in *S. aureus* (Bergdoll et al., 1965, 1971, 1973; Casman et al., 1967; Marrack and Kappler, 1990), advancements in the area of molecular biology during the 1980s led to the identification of a new set of genes encoding closely-related proteins with superantigenic and emetic activities (Table 1). This sudden increase in the number of described SEs spurred a move to standardize their nomenclature (Lina et al., 2004). Only enterotoxins with demonstrated emetic potential in monkeys were designated “SE,” whereas enterotoxins that failed to do so or have not been evaluated in non-human primate models of emesis are designated enterotoxin like (SE*l*-) toxins (Table 1). The only exception to this rule is Toxic Shock Syndrome Toxin-1 (TSST-1), which was originally designated SEF (Bergdoll et al., 1981; Reiser et al., 1983). This toxin's apparent lack of emetic activity, possibly due it being less stable than other SEs (Edwin and Kass, 1989), prompted the name change to TSST-1, which has remained in place ever since. Joining TSST-1, SE*l*J is the only other tested SE that is non-emetic (Munson et al., 1998; Orwin et al., 2001, 2002). SE*l*X, SE*l*U, SE*l*W, SE*l*V, and SE*l*Y have yet to be tested for emetic activity in non-human primates.

**Table 1:** Emetic and superantigenic activities of staphylococcal enterotoxins.

**Enterotoxin**	**Genetic element**	**Superantigenic activity**	**Emetic activity**		**Type**	**Phylogenetic group**
			**Monkey**	**Shrew**		
SEA	Prophage	Yes	Yes (Bergdoll et al., 1965)	Yes (Hu et al., 2003)	Classical	SEA
SEB	Chromosome, SaPI, plasmid (pZA10)	Yes	Yes (Bergdoll et al., 1965)	Yes (Hu et al., 2003)	Classical	SEB
SEC1	SaPI	Yes	Yes (Schlievert et al., 2000)	nd^1^	Classical	SEB
SEC2	SaPI	Yes	Yes (Bergdoll et al., 1965)	Yes (Hu et al., 2003)	Classical	SEB
SEC3	SaPI	Yes	Yes (Reiser et al., 1984)	nd	Classical	SEB
SED	Plasmid (pIB485)	Yes	Yes (Igarashi, 1972)	Yes (Hu et al., 2003)	Classical	SEA
SEE	Prophage	Yes	Yes (Bergdoll et al., 1971)	Yes (Hu et al., 2003)	Classical	SEA
SEG	*egc*1, *egc*2, *egc*3, *egc*4	Yes	Yes (Munson et al., 1998)	Yes (Hu et al., 2003)	New	SEB
SEH	Transposon (MGEmw2/mssa476 *seh*/D*seo*)	Yes	Yes (Su and Wong, 1995)	Yes (Hu et al., 2003)	New	SEA
SEI	*egc*1, *egc*2, *egc*3	Yes	<100 μg/kg (Munson et al., 1998)	Yes (Hu et al., 2003)	New	SEI
SE*l*J	Plasmid (pIB485, pF5)	Yes	nd	nd	New	SEA
SEK	Prophages, SaPI1, SaPI3, SaPI5, SAPIbov1	Yes	Yes (Omoe et al., 2013)	Yes (Ono et al., 2017)	New	SEI
SEL	Prophages, SaPIn1, SaPIm1, SaPImw2, SAPIbov1	Yes	Yes (Omoe et al., 2013)	Yes (Ono et al., 2017)	New	SEI
SEM	*egc*1, *egc*2	Yes	Yes (Omoe et al., 2013)	Yes (Ono et al., 2017)	New	SEI
SEN	*egc*1, *egc*2, *egc*3, *egc*4	Yes	Yes (Omoe et al., 2013)	Yes (Ono et al., 2017)	New	SEA
SEO	*egc*1, *egc*2, *egc*3, *egc*4, transposon	Yes	Yes (Omoe et al., 2013)	Yes (Ono et al., 2017)	New	SEA
SEP	Prophage (Sa3n)	Yes	Yes (Omoe et al., 2013)	Yes (Omoe et al., 2005)	New	SEA
SEQ	Prophage, SaPI1, SaPI3, SaPI5	Yes	Yes (Omoe et al., 2013)	Yes (Hu et al., 2017)	New	SEI
SER	Plasmid (pIB485, pF5)	Yes	<100 μg/kg (Ono et al., 2008)	<100 μg/kg (Ono et al., 2008)	New	SEB
SES	Plasmid (pF5)	Yes	<100 μg/kg (Ono et al., 2008)	<100 μg/kg (Ono et al., 2008)	New	SEA
SET	Plasmid (pF5)	Yes	<100 μg/kg (Ono et al., 2008)	<100 μg/kg (Ono et al., 2008)	New	SE*l*X
SEU	*egc*2, *egc*3	Yes	nd	nd	New	SEB
SE*l*W (SE*l*U2)	*egc*4	Yes	nd	nd	New	SEB
SEV	*egc*4	Yes	nd	nd	New	SEI
SE*l*X	Chromosome	Yes	nd	nd	New	SE*l*X
SE*l*Y	Chromosome	Test cell-dependent	nd	Yes (Ono et al., 2015)	New	SE*l*X

*nd, not demonstrated*.

## The SE genes are distributed across a variety of different genomic locations

When considering the locations of the enterotoxin genes, *selx* (Wilson et al., 2011) and *sely* (Ono et al., 2015) are unique as they are found exclusively on the genome. The *selx* gene can be found in ~95% of *S. aureus* strains, whereas *sely* appears less frequently and has only been detected in a handful of strains thus far. In contrast, the other enterotoxin genes are sometimes found alone, but more commonly in groups, on a variety of large mobile segments of DNA called mobile genetic elements (MGEs) (Fraser and Proft, 2008; Argudin et al., 2010). These MGEs include prophages, plasmids, transposons, *S. aureus* pathogenicity islands (SaPIs), and the enterotoxin gene clusters (*egc*) (Table 1) (for a review on staphylococcal MGEs see, Malachowa and DeLeo, 2010). The *egc* locus is home to an operon of genes encoding SEG, SEI, SEM, SEN, SEO, and two pseudogenes, φ*ent1* and φ*ent2* (Jarraud et al., 2001; Monday and Bohach, 2001). Deletion, duplication and recombination events within this cluster make it a major hub for the generation of new types of SEs and variants (Letertre et al., 2003b; Thomas et al., 2006). The acquisition of MGEs generally has a significant impact on core genomes by causing striking differences in genome size and structure. In *S. aureus*, a comparison of the presence of SE genes from several major lineages shows that SE gene composition is strongly linked to specific genetic backgrounds, emphasizing the importance of vertical transmission, rather than horizontal transmission, of SE-encoding MGEs (Goerke et al., 2009). Around 80% of *S. aureus* isolates, including commensal, clinical, and food-poisoning isolates, carry an average of 5–6 SE genes (Jarraud et al., 2001; Baba et al., 2002; Becker et al., 2003; Holtfreter et al., 2004, 2007; Hait et al., 2014; Lv et al., 2014; Umeda et al., 2017).

## The enterotoxins can be further separated based on nucleotide and amino acid sequences

The 24 currently identified SEs and SE*l*s, can be further separated into several evolutionary groups based on a comparison of their nucleotide and amino acid sequences; the SEA group (SEA, SED, SEE, SE*l*J, SEH, SEN, SEO, SEP, SES), the SEB group (SEB, SECs, SEG, SER, SE*l*U, SE*l*W, previously known as SE*l*U2), the SEI group (SEI, SEK, SEL, SEQ, SEM, SE*l*V), and the SE*l*X group (TSST-1, SET, SE*l*X, SE*l*Y and members of another group of staphylococcal exotoxins called superantigen-like (SSL) toxins) (for reviews, see Fraser and Proft, 2008; Ono et al., 2015) (Table 1). A fifth group, which is not produced by staphylococci, but only represented by a group of functionally and structurally similar superantigenic toxins produced by streptococci, will not be discussed further.

The presence or absence of two specific structural features predominantly defines the superantigenic and enterotoxigenic properties of the SEs and explains differences in activity between the evolutionary groups. First, enterotoxins belonging to the SE*l*X and SEB groups only possess one low affinity α-chain major histocompatibility complex (MHC) II binding site, whereas enterotoxins from the SEA and SEI groups contain one low affinity α-chain MHC II and a second, high affinity β-chain MHC II binding site, which generally equates to superior superantigenic activity (Kozono et al., 1995). Additionally, differences in amino acid composition have given rise to variants of SEB (Kohler et al., 2012), SEC (Bohach and Schlievert, 1987; Couch and Betley, 1989; Marr et al., 1993), SED (Johler et al., 2016), SEG, SEI (Abe et al., 2000; Blaiotta et al., 2004), SEK (Aguilar et al., 2014), SEM, SEN, SEO, SE*l*U, and SE*l*V (Letertre et al., 2003b; Collery et al., 2009). Compared to the parent toxins, variants of SEB (Kohler et al., 2012) and SEC (Deringer et al., 1997) demonstrate altered species tropism or reduced superantigenic activities. The production of these mutations in SEs may be part of a broader strategy of *S. aureus* to adapt to different host species (Marr et al., 1993; Edwards et al., 1997; Johler et al., 2016).

Second, a separate and distinct loop comprising 9–19 varying amino acids flanked by 2 cysteine residues creating a disulfide bridge, was originally thought to be an essential feature of emesis-inducing SE members from the SEA and SEB evolutionary groups. However, mutational analyses of that loop demonstrated that only the disulfide bond between the two cysteine residues, rather than the loop itself, was required for emesis (Hovde et al., 1994). These data are consistent with experiments demonstrating that SEs that lack the loop can still induce emesis in primates (Omoe et al., 2013), leading to the conclusion that there are additional unidentified emesis-associated structural determinant(s) in the SEs.

## *S. aureus* has a complex network of regulatory pathways to control toxin production

*S. aureus* responds to changes in the environment using a combination of quorum-sensing (QS) (Waters and Bassler, 2005) and other two-component systems (TCS), of which at least 16 have been discovered in *S. aureus* to date (Haag and Bagnoli, 2016), as well as many trans-acting regulatory proteins (Bronner et al., 2004). *S. aureus* relies on these systems to quickly make changes in the regulation of genes associated with important physiological features, including drug resistance, metabolism, immune evasion, and virulence. Each system can directly or indirectly control the transcription of specific sets of genes. However, the regulation of one gene may be influenced by multiple systems, leading to additional layers of regulation.

The accessory gene regulator (Agr) QS system, which is activated at high cell densities, is comprised of two transcriptional units transcribed in opposing directions; RNAII, which codes for four genes (*agrA, agrB, agrC*, and *agrD*) (Novick et al., 1995) and RNAIII, a regulatory RNA. These transcripts are controlled by the promoters P2 and P3, respectively. AgrD, which contains the sequence for the autoinducing peptide (AIP), is processed and exported out of the cell by the combined actions of the membrane-associated export protein, AgrB (Ji et al., 1995, 1997; Mayville et al., 1999) and a type I signal peptidase, SpsB (Kavanaugh et al., 2007). AIP acts as the ligand for the membrane bound histidine kinase, AgrC, leading to the phosphorylation of AgrA (Ji et al., 1995; Lina et al., 1998). Activated ArgA binds to the P2 and P3 promoters, resulting in the perpetuation of a positive feedback loop (Koenig et al., 2004).

Expression of *agr* is affected by various trans-activing regulators, such as the Sar family of regulatory proteins, (SarR, SarS, SarT, SarU, SarX, SarZ, SarV, MgrA, and Rot) (Cheung and Projan, 1994; Heinrichs et al., 1996; Cheung et al., 2008), σB (Lauderdale et al., 2009), and SrrAB (Staphylococcal respiratory response AB) (Yarwood et al., 2001; Pragman et al., 2004). Additionally, σB and Rot can affect another important two-component system called SaeRS (Li and Cheung, 2008; Kusch et al., 2011). Importantly, all these regulatory elements respond to various environmental stresses and stimuli; the SaeRS (*S. aureus* exoprotein expression) system responds to membrane attack by antimicrobial molecules produced by the innate host defense (Novick and Jiang, 2003; Kuroda et al., 2007; Geiger et al., 2008; Cho et al., 2015), SarA largely responds to changes in microenvironments (Cheung et al., 2004), σB responds to high temperature, catabolites, alkaline pH, high salinity (Betley et al., 1992; Wu et al., 1996; Kullik and Giachino, 1997; Kullik et al., 1998; Pané-Farré et al., 2006), whereas the SrrAB system has been shown to be particularly crucial for bacterial growth under anaerobic and hypoxic conditions (Yarwood et al., 2001; Pragman et al., 2007; Kinkel et al., 2013; Mashruwala and Boyd, 2017). Lastly, Rot, the global gene regulator (Saïd-Salim et al., 2003) is negatively regulated by RNAIII through an antisense mechanism (Geisinger et al., 2006; Boisset et al., 2007).

## Regulation of the classical enterotoxins

**Figure 1:**
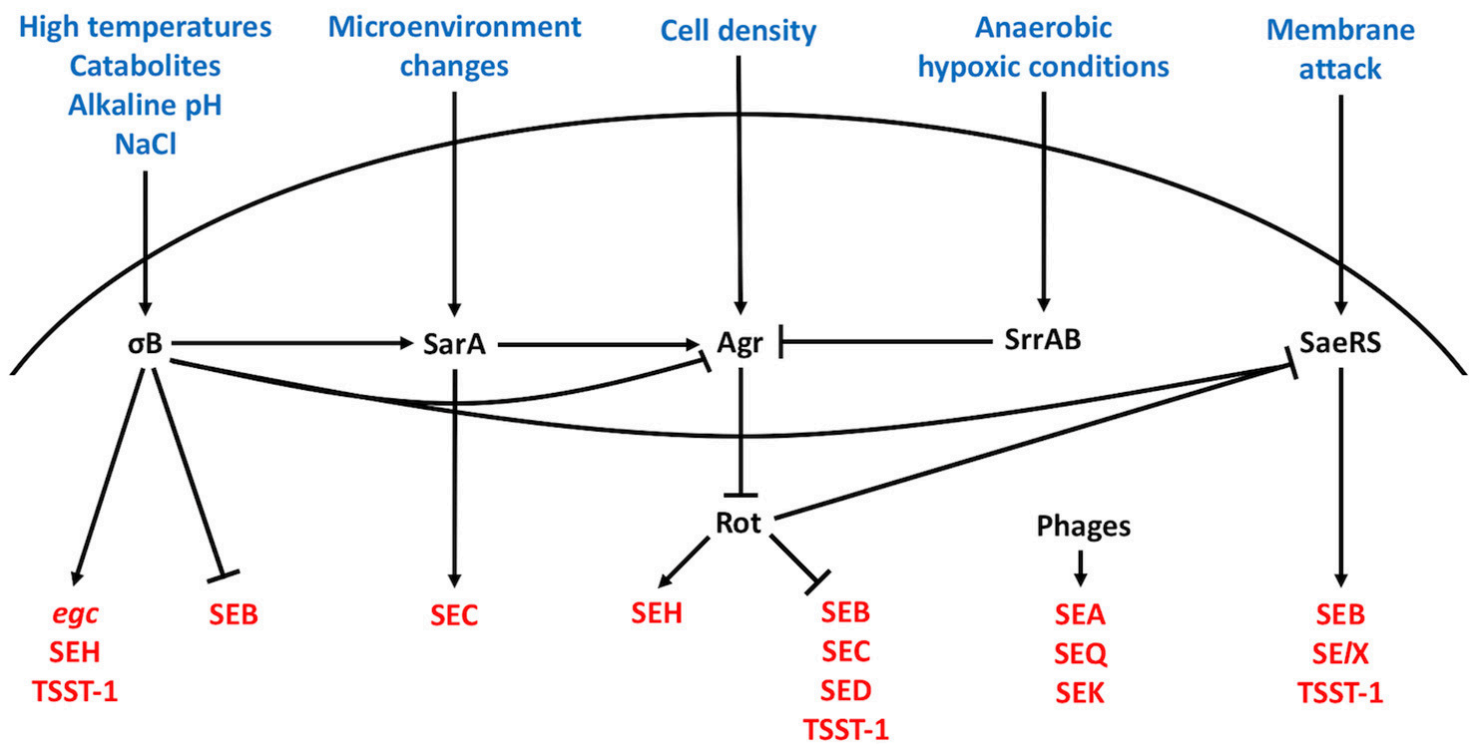
Regulation of staphylococcal enterotoxins. Harsh bacterial growth conditions, changes in the bacterial microenvironment, high cell density, hypoxia, and membrane changes direct enterotoxin expression through the alternative sigma factor, SarA protein family, Agr quorum sensing system, SrrAB protein, and SaeRS two-component system, respectively. The excitatory and inhibitory action of these systems on the other regulators and enterotoxins are summarized. Arrowheads represent upregulation and bars downregulation.

It has been described early that there is unequal distribution of SE-associated MGEs among *S. aureus* isolates, and that thus, the host background has profound influences on enterotoxin production (Gaskill and Khan, 1988; Compagnone-Post et al., 1991). Surprisingly, our understanding of how the enterotoxins are regulated is still rather incomplete, but we do know that enterotoxin regulation is strongly dependent on the regulatory systems described above (Figure 1). Several Agr-controlled staphylococcal toxins, such as alpha-toxin (Morfeldt et al., 1995) and the family of phenol-soluble modulins (PSMs) (Queck et al., 2008) are produced between the early logarithmic and stationary phases. Early observations showing that the production of SEB (Czop and Bergdoll, 1974; Gaskill and Khan, 1988; Derzelle et al., 2009), SEC (Otero et al., 1990; Regassa et al., 1991), and SED (Bayles and Iandolo, 1989) also occurred between the exponential to stationary phases of bacterial growth (Gaskill and Khan, 1988; Regassa et al., 1991; Zhang and Stewart, 2000) suggested that they could be regulated by Agr. Indeed, isogenic *S. aureus agr* mutants showed significant decreases in SEC and SED production compared to the wild-type strain (Regassa et al., 1991). However, it was later shown that SEB, SEC, and SED is regulated indirectly by other factors. For instance, Agr-dependent regulation of SEB, SEC, and SED occurs via RNAIII-dependent inhibition of Rot (Regassa and Betley, 1993; Tseng et al., 2004; Tseng and Stewart, 2005). In addition to Rot, SEB is also negatively regulated by σB (Ziebandt et al., 2001, 2004; Pané-Farré et al., 2006; Rogasch et al., 2006).

In contrast, the production of bacteriophage-associated SEA is generally constitutive (Thomas et al., 2007), although *S. aureus* strains with distinct high and low SEA expression patterns have been described (Borst and Betley, 1994; Wallin-Carlquist et al., 2010). Since the expression pattern of SEA was found to be different from that of SEB, SEC and SED, it was postulated and confirmed that SEA is regulated independently of Agr (Tremaine et al., 1993). The production of SEA was later discovered to be closely tied to the phage's life cycle (Cao et al., 2012) and to be inducible by bacterial stress (Zeaki et al., 2015).

## Regulation of the newer enterotoxins

Information surrounding the regulation of the newer enterotoxins is only beginning to emerge. Unlike most of the classical enterotoxins, it appears that the regulation of several newer enterotoxins including SE*l*J (Zhang et al., 1998) and SEH (Lis et al., 2012), is Agr-independent. The expression of SFP-associated SEH, which is produced predominantly in the late exponential phase of bacterial growth (Sakai et al., 2008; Lis et al., 2012), was recently shown to be positively regulated by Rot, via direct binding to the *seh* promoter (Sato'o et al., 2015), σB (Kusch et al., 2011), several Sar homologs, and SaeR (Sato'o et al., 2015). Moreover, SaeRS appears to have a positive impact on SE*l*X (Langley et al., 2017) and TSST-1 (Baroja et al., 2016) expression. In contrast, the production of enterotoxins encoded in the *egc* operon (SEG, SEI, SEM, SEN, SEO, and SE*l*U) is highest in the earliest stages of exponential growth (Grumann et al., 2008) and dependent on σB (Kusch et al., 2011). Interestingly, one study showed that SEK production is dependent on the presence of SEB (Aguilar et al., 2014), whereas SEK and SEQ, which are also found on *sea*-associated phages, can be transcriptionally induced by mitomycin C (Sumby and Waldor, 2003). Taken together, the SEs are regulated by multiple regulatory elements that respond to a variety of different environmental signals. Likely, the delicate balance in enterotoxin expression facilitated by these regulatory elements has a profound impact on the commensal and pathogenic lifestyles of *S. aureus*.

## Which staphylococcal enterotoxins contribute to SFP?

To control staphylococcal food poisoning and ensure food safety, the roles of both new and classical SEs must be considered. Although a wide variety of SE detection methods have been developed (Table 2), molecular detection of SE genes remains the most common method used for investigating the possible contribution of SEs toward SFP. Molecular studies spanning the last two decades have shown that *egc-*encoded genes (*seg, seh, sei*, or *selj*) are readily detected in *S. aureus* food poisoning isolates around the world (Blaiotta et al., 2004; Grumann et al., 2008; Yan et al., 2012; Viçosa et al., 2013; Chao et al., 2015; Johler et al., 2015; Cheng et al., 2016; Song et al., 2016; Shen et al., 2017; Umeda et al., 2017). Additionally, the detection of non *egc*-encoded enterotoxin genes, such as transposon associated-*seh* (McLauchlin et al., 2000; Ikeda et al., 2005; Jørgensen et al., 2005), plasmid-associated *ser* (Wattinger et al., 2012) and SaPI-associated *seq* (Chiang et al., 2008; Alibayov et al., 2014; Lv et al., 2014; Hu et al., 2017) suggest a role of these newer SEs in SFP.

**Table 2:** A summary of detection strategies for staphylococcal enterotoxins.

**Method of detection**	**Description**	**Comments**	**References**
Animals	Emesis in kittens		Fulton, 1943
	Emesis in house musk shrews	Animal testing is generally labor intensive and expensive	Hu et al., 1999
	Emesis in dogs	Inter-animal and species differences can affect results	Kocandrle et al., 1966
	Emesis in pigs and piglets	Low sensitivity in some species	Taylor et al., 1982; Van Gessel et al., 2004
	Emesis in ferrets		Wright et al., 2000
	Emesis in monkeys		Bergdoll et al., 1965; Sugiyama and Hayama, 1965
	Skin test in guinea pigs		Scheuber et al., 1983
	Mouse, rat, and rabbits^a^		Horn et al., 2013
Serological testing	Gel diffusion/agglutination tests	Semi-quantitative. Lack in specificity and sensitivity have prevented these assays from being employed for routine detection of SEs	Read et al., 1965; Salomon and Tew, 1968
Immunoassays	Colorometric	Colorometric method is most commonly used for SE protein detection	Saunders and Bartlett, 1977
	Fluorescent (including Quantum dots and Lanthanide ion chelate-doped nanoparticles)		Tempelman et al., 1996; Goldman et al., 2002
	Chemiluminescent	All methods are highly sensitive and specific and provide low background signals	Luo et al., 2006
Coupled immunoassays	Electrochemiluminescent	Easy and rapid to operate, low costs Can detect presence of over a wide linear range and in complex samples	Kijek et al., 2000; Sun et al., 2010
	Surface plasmon resonance		Rasooly and Rasooly, 1999; Nedelkov et al., 2000
	Surface-Enhanced Raman Scattering		Pekdemir et al., 2012
	Electrochemical mass		Harteveld et al., 1997
Molecular	Colony blot hybridization	Simultaneous detection of several SE genes with different primers	Neill et al., 1990
	Polymerase chain reaction (PCR)		Wilson et al., 1991
	Multiplex PCR	Fast and can be applied to detect SE genes in most kinds of food	Shylaja et al., 2010
	Real-time PCR	Methods do not detect the presence of protein toxins	Letertre et al., 2003a
	Reverse-transcriptase PCR		Matsui et al., 1997
	Loop-mediate isothermal amplification (LAMP)		Nkouawa et al., 2009
Chromatography	Liquid chromatography tandem-mass spectrometry (LC-MS/MS	Does not require the isolation of toxins from food. Highly sensitive. However, samples with high protein levels may suppress electrospray.	Kientz et al., 1997
	Liquid chromatography Electrospray ionization mass spectrometry (LC-ESI/MS)		Callahan et al., 2006
			
Aptamer-based bioassays	DNA and RNA	Highly specific, comparable to antibodies. Easily produced by chemical synthesis, high purity and easily modified with chemical tags. Can be coupled with other techniques.	Bruno and Kiel, 2002
	Peptide		Soykut et al., 2008
	Molecularly imprinted polymers		Gupta et al., 2011

a *No emetic reflexes observed in these species*.

While PCR is an invaluable tool, confirmation of the physical presence of toxin in food products suspected of contamination is needed to clearly verify their contribution to SFP. The immunological detection of the 5 classical SEs has helped to establish SEA as the top contributor (~80%) to SFP outbreaks (Pinchuk et al., 2010; Hennekinne et al., 2012), followed by SED, SEB, SEC, and SEE (Hu and Nakane, 2014). In contrast, due to the lack of sensitive detection methods, it has been impossible to draw such conclusions for the newer SEs. However, a steadily increasing number of immunological assays for the non-classical enterotoxins, such as SEG (Nagaraj et al., 2016), SEH (Su and Wong, 1996), SEI (Zhao et al., 2016b), SEK (Aguilar et al., 2014), SEM (Zhao et al., 2017), and SEQ (Hu et al., 2017) have been developed within the last decade. They indicated that one or more of the newer enterotoxins are potential causes of SFP outbreaks. Although few studies have examined the physical presence of multiple enterotoxins, it is most likely that multiple SEs contribute to SFP. The expansion of existing multiplex assays (Liang et al., 2015; Adhikari et al., 2016) would be the most efficient strategy to detect all SEs simultaneously. However, each platform has its advantages and disadvantages (Table 2; Wu et al., 2016 for review). An ideal platform to detect all SEs would have high sensitivity, low cross-reactivity, and universal adaptability. Although creating such a system is not impossible, it would be an extremely difficult task, requiring considerable resources, and vigorous testing.

## Humans and livestock are major reservoirs for the transmission of enterotoxin-producing *S. aureus*

*S. aureus*, a natural colonizer of humans, can be found on the skin (primarily on the hands, chest, and abdomen), gastrointestinal (GI) tract (Ridley, 1959; Armstrong-Esther, 1976; Wertheim et al., 2005), and nasopharyngeal cavities (Williams, 1963). All these sites represent possible reservoirs for the distribution of *S. aureus* causing human disease. Persistent colonization of the anterior nares with *S. aureus*, which currently is estimated to be around 20–30% of the population (Verhoeven et al., 2014), is believed to be the most important risk factor for infection, especially regarding health-care associated diseases (Von Eiff et al., 2001). While colonization of the GI tract by *S. aureus* has received significantly less attention, recent studies emphasize its underappreciated role in the association with and transmission of *S. aureus* disease (Nowrouzian et al., 2011, 2017; Senn et al., 2016; Gagnaire et al., 2017). With regards to SFP, studies investigating the presence of enterotoxin genes in *S. aureus* isolates sampled from the nose (Nashev et al., 2007; Collery et al., 2009; Wattinger et al., 2012; Ho et al., 2015) and gut (Lis et al., 2009; Shin et al., 2016) indicate that these two sites are important sources of enterotoxin-producing *S. aureus*.

*S. aureus* is particularly renowned for its ability to acquire and develop resistance to multiple antibiotics, which is a key factor contributing to the difficultly of treating infections caused by this pathogen. A majority of *S. aureus* infections are caused by methicillin-resistant strains (MRSA), which, historically, have been associated with disease in hospitalized patients in a variety of public healthcare settings [hospital-associated (HA)-MRSA]. However, in the early 1990s, a new breed of genetically distinct MRSA strains started to appear in the community [community-associated (CA)-MRSA] (Otto, 2010). Compared to the HA-MRSA strains, CA-MRSA strains are exceptionally pathogenic (Chambers, 2001; Cameron et al., 2011) because of the enhanced production and acquisition of a broad set of virulence factors that contribute to fitness, colonization and virulence (Otto, 2012). Additionally, MRSA infections in the community can be caused by strains initially associated with livestock [livestock-associated MRSA (LA-MRSA)] (Huijsdens et al., 2006; Lewis et al., 2008; Nemati et al., 2008). For instance, carriage, or infections caused by *S. aureus* in dairy cattle (e.g., mastitis) can lead to the contamination of dairy products and raw meat. In particular, unprocessed foods hold a substantial risk for the introduction of resistant microbes into the food chain, which can have a considerable economic impact, especially in countries with industrialized dairy sectors (Le Loir et al., 2003). Interestingly, epidemiological studies have indicated that LA-MRSA isolates belong to genetic lineages different from their HA- and CA-MRSA counterparts (for detailed reviews, see Fluit, 2012; Cuny et al., 2015; Smith, 2015) and harbor unique genes that are essential for host adaptation (Lowder et al., 2009; Guinane et al., 2010; Price et al., 2012).

Unsurprisingly, several recent studies reported high levels of multiple antibiotic resistance in LA-MRSA (Kérouanton et al., 2007; Ge et al., 2017; Sahibzada et al., 2017; Abdi et al., 2018; Suleiman et al., 2018), but unlike other enteric pathogens, such as *Salmonella* and *E. coli*, for which antimicrobial resistance can impose serious health risks in humans (Doyle, 2015), antibiotic resistance in HA-, CA-, or LA-MRSA isolates had little influence on the ability of *S. aureus* to cause SFP (Sergelidis and Angelidis, 2017). These observations are consistent with the notion that SFP is not a disease that is typically treated with antibiotics, since the enterotoxin-driven manifestation of SFP can progress in the absence of bacteria.

## Mechanisms underlying enterotoxin-induced emetic and diarrheal activity

**Figure 2:**
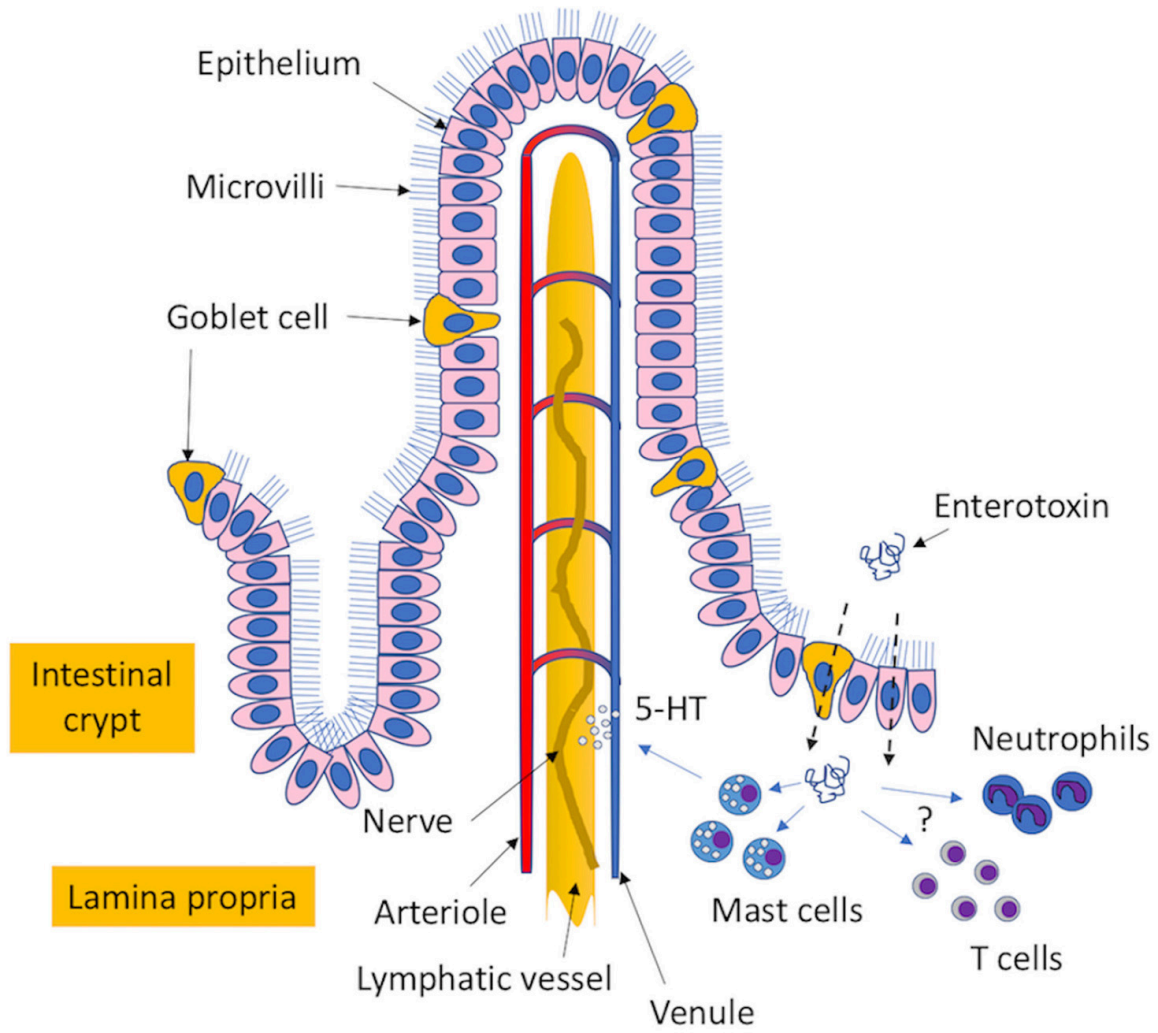
Proposed mechanism of enterotoxin-induced emesis. The enterotoxins transit through mucus-expelling goblet cells and epithelial cells in the intestinal epithelium to reach the lamina propria. Here, the enterotoxins can interact with mast cells to induce the release of 5-hydroxytryptamine (5-HT/serotonin precursor), which interacts with the vagus nerve to cause an emetic response. Additional cellular targets that may have possible roles in the induction of enterotoxigenic disease include different types of T cells and neutrophils.

Progress in understanding the molecular mechanisms underlying the enterotoxigenic effects of the SEs has been hampered by a lack of relevant animal models. Small rodents, such as mice and rats, are non-emetic and generally less susceptible to the enterotoxigenic effects of the SEs (Bergdoll, 1988) whereas non-human primates, which are considered the gold standard for testing the emetic activity of enterotoxins, are costly and riddled with complex requirements in animal care and husbandry. However, the house musk shrew, *Suncus murinus*, was recently identified as a suitable animal model and an alternative to using monkeys (Hu et al., 2003). Studies in the shrew confirmed that a network of branched connections linking multiple organs of the body with the brain, called the vagus nerve, was an essential element for SE-induced emesis, recapitulating earlier observations from monkeys (Sugiyama and Hayama, 1965). Further studies in shrews revealed that the MHC II-independent release of 5-hydroxytryptamine (5-HT/Serotonin) from mast cell granules by SEs was crucial for SE-induced emesis (Ono et al., 2012). Other agonists involved in the emetic response have also been reported (Scheuber et al., 1987; Alber et al., 1989; Jett et al., 1990). In addition to mast cells, the SEs appear to have an affinity for epithelial cells (Hamad et al., 1997; Shupp et al., 2002; Danielsen et al., 2013; Zhao et al., 2016a) and goblet cells (Hirose et al., 2016). Unlike mast cells, SEs use epithelial cells (Danielsen et al., 2013) and mucus-producing goblet cells (Hirose et al., 2016) as gateways in order to traffic across the intestinal epithelia to reach other final targets. Importantly, the movement of enterotoxins through epithelial cells is thought to be a glycolipid-dependent transcytosis process that may be facilitated in the presence of other *S. aureus* virulence determinants (Edwards et al., 2012). Interestingly, a conserved stretch of 10-amino-acid peptides, located within the longest alpha-helical chain between the A and B domains of the enterotoxins, is an important structural determinant that promotes translocation (Shupp et al., 2002; Figure 2).

In contrast to strong induction of emesis, the clinical symptoms of diarrhea are oftentimes less apparent in SFP, which may be in part due to the inability of some SEs, such as SEA and SEC, to cause fluid exudation and dilation of the intestinal segments (Maina et al., 2012). However, the symptoms of diarrhea sometimes observed with SEB intoxication may be due to the inhibition of water and electrolyte reabsorption in the small intestine (Sullivan, 1969; Sheahan et al., 1970). To this date, exactly how the SEs cause diarrhea is still far from understood. For a detailed review on other aspects of SE-induced emesis, see (Hu and Nakane, 2014).

## The superantigenic activities of the enterotoxins

The molecular details underlying the superantigenic activity of the SEs have been dissected by numerous X-ray crystallography, structural and mutational analyses. Unlike with conventional antigens, the non-specific activation of T cells by SEs occurs independently of antigen processing and presentation to the T cells by antigen-presenting cells (APCs). Instead, SEs act as a bridge between APCs and T cells. In the majority of cases, SEs first bind to the MHC class II molecules found on APCs and coordinate binding to one or more variable beta (Vβ) chain(s) of T-cell receptors (TCRs) (Kappler et al., 1989; White et al., 1989; Choi et al., 1990; Jarraud et al., 2001). However, these molecular interactions are not exclusive and other receptors have been described to be involved. For instance, the variable alpha (Vα) chain can be targeted by SEH (Saline et al., 2010). Moreover, maximal superantigenic activity of SEB is dependent on additional co-stimulatory receptors, CD28 and B7-2, on T cells and APCs, respectively (Arad et al., 2011; Levy et al., 2016). Interestingly, the same CD28 binding site can be found on other SEs, such as SEA and TSST-1 (Arad et al., 2011). Regardless of the mechanism of cross-linking, characteristic for SE activity is a polyclonal activation of a large pool of CD4^+^ and CD8^+^ T cells (~20% of the total T cells) (Marrack et al., 1990; Miethke et al., 1992; Leder et al., 1998) followed by a massive release of an assortment of T helper 1 (Th1) cytokines, such as tumor necrosis factor (TNF) α, interleukin 1 (IL-1), IL-2, and interferon (IFN) γ (Carlsson et al., 1988; Tiedemann and Fraser, 1996), all of which contribute to the SE superantigenic effect (for a detailed reviews, see Krakauer, 2013; Krakauer et al., 2016).

## The enterotoxins are immunomodulators of multiple immune cell types

The superantigenic and enterotoxigenic activities of the SEs are the best studied mechanisms underlying their pathogenicity. However, recent studies show that the SEs possess functions in addition to these conventional activities. For example, both TSST-1 and SE*l*X (Wilson et al., 2011) show similarity to another family of staphylococcal exotoxins, called the staphylococcal superantigen-like (SSL) toxins (reviewed in Fraser and Proft, 2008). Although the SSL toxins lack the ability to induce Vβ-specific T-cell proliferation, they have diverse roles in immune evasion, including the ability to interfere with complement activation and neutrophil function (reviewed in Langley et al., 2010). Recently, it was discovered that SE*l*X has a unique sialic acid-binding motif. This motif allows SE*l*X to interact with adhesion molecules on neutrophils involved in immune recognition and cell activation (Langley et al., 2017; Tuffs et al., 2017). Importantly, the ability of SE*l*X to bind neutrophils, which are considered the first line of defense against *S. aureus* (Spaan et al., 2013), was crucial for disease progression in a rabbit model of necrotizing pneumonia. Together, these studies describe an unusual member of the SE family that has both superantigenic and SSL functions.

Neutrophils are the latest among a growing list of immune cell types recognized to be targeted directly or indirectly by the SEs. Others include γδ T cells (Maeurer et al., 1995; Morita et al., 2001), invariant natural killer T (*i*NKT) cells (Rieder et al., 2011; Hayworth et al., 2012), B cells (Stohl et al., 1994), mast cells (Scheuber et al., 1987; Lotfi-Emran et al., 2017), and mucosa-associated invariant T (MAIT) cells (Shaler et al., 2017). Activation of these cell types by SEs can have a considerable impact on the immune system, which may lead to non-conventional overstimulation of the immune system, as exemplified by B cell proliferation and differentiation into plasma cells (Stohl et al., 1994). Additionally, excessive inflammation, as a result of the direct activation of *i*NKT cells and γδ T cells, can cause the production of SE-associated inflammatory disease in the lungs (Rieder et al., 2011) and systemic infection, as demonstrated in mouse infection models (Szabo et al., 2017).

In contrast to the overstimulation of the immune response by SEs, the activation of MAIT cells appears to have the opposite effect (Shaler et al., 2017). MAIT cells have significant roles in innate host defense against a variety of pathogens (Napier et al., 2015). Notably, the activation of MAIT cells by SEs was shown to be induced in a TCR-independent manner (Shaler et al., 2017). While direct activation of the MAIT cells by SEs could not be excluded, MAIT cell activation was mediated mostly by IL-12 and IL-18 released from the direct activation of conventional T cells by SEs (Shaler et al., 2017). Following a period of hyper-activation, these MAIT cells rapidly undergo exhaustion and are unable to respond further, leaving behind a suppressed and severely crippled arc of innate host defense.

## Could enterotoxicity be dependent on T-cell immunomodulation?

Whether the superantigenic function is needed for the enterotoxigenic activity of the SEs is an interesting question. Shock and fever, hallmarks of superantigen-induced disease, is generally low or absent in patients with SFP (Dinges et al., 2000), arguing against the activation of a systemic immune response. However, it was shown that 5 times more of an SEA protein derivative, which lacked superantigenic but retained emetic activity, was required to induce emesis in a monkey model compared to unaltered SEA (Hoffman et al., 1996). This observation implies that both superantigenic and enterotoxicity activities are likely needed for a maximal emetic response.

Another aspect of immune interaction that may need to be further investigated is the potential role of T cells in SE enterotoxic activities. MAIT cells for example, which have been shown to have a protective role against GI bacterial disease (Powell and Macdonald, 2017; Salerno-Goncalves et al., 2017), represent ~10% of intestinal T cells (Treiner et al., 2003; Dusseaux et al., 2011) and ~50% of T cells in the intestines express γδ TCRs (Carding and Egan, 2002). Furthermore, γδ T cells that are present in the gut mucosa play an important role in mucosal immunity (Agace, 2008). Additionally, given that the SEs are highly potent at very low concentrations, enhanced expression of SEs may not be essential for the advancement of SE-mediated disease. In fact, when regulatory T cells (Tregs) are stimulated with lower concentrations of SEC, an immunosuppressed phenotype can be induced that may directly benefit *S. aureus* colonization and disease progression (Lee et al., 2017). In the healthy gut, Tregs play a crucial role in the maintenance of intestinal homeostasis by controlling inappropriate immune responses (Luu et al., 2017). Therefore, it is tempting to speculate that the combined targeting of MAIT cells, γδ T cells and Tregs in the gut by SEs may promote the pathogenesis of SFP. Whether MAIT, γδ T cells, and Tregs play any roles in SFP requires much more detailed investigation.

## Conclusions

Although the classical enterotoxins have historically been considered the predominant contributors to SFP, a number of molecular studies suggest that many of the newer SEs also have a prominent role. However, in order to better determine which SEs are responsible for SFP, it is best for studies investigating SFP outbreaks to employ methods that can detect all SE genes as well as the physical presence of toxin in suspected contaminated foods. The ability to culture and accurately characterize SFP-causing *S. aureus* will significantly help understand true incidence and prevalence of SFP. It should also be noted that the inability to detect SEs in contaminated foods does not exclude that they contribute to SFP. Therefore, it is just as vital that we have a deeper understanding of what promotes SE production, especially in food environments. While it is accepted that multiple regulatory networks can have a significant impact on enterotoxin expression, it remains poorly understood how specific enterotoxins, especially the newer enterotoxins, are regulated.

In this review, we also provided an overview of the molecular mechanisms that contribute to SFP. Yet, compared to what we know about staphylococcal superantigen-associated disease, our comprehension of the structural elements and mechanisms by which SEs induce SFP has remained limited, especially considering that SFP is a common disease that continues to affect millions worldwide. A key gap in our knowledge is whether the superantigenicity of the SEs plays a pathogenic role in SFP. There is evidence that suggests that the manifestation of SFP does not solely rely on the enterotoxic function of SEs. Furthermore, we highlighted that different immune and non-immune cell types are susceptible to immunomodulation by the SEs. Any possible interaction between the SEs and these cell types, especially in the gut environment, is worth exploring. Overall, the molecular details involved in SE-mediated enterotoxigenic disease are slowly being uncovered; however, many basic questions remain. Future challenges therefore will consist of deciphering the series of events that lead to disease and whether there are other key cellular players, and identifying an appropriate animal model that is amenable to genetic manipulation.

## Author contributions

EF, GC, and MO: contributed to the drafting of the manuscript and approved the final version.

### Conflict of interest statement

The authors declare that the research was conducted in the absence of any commercial or financial relationships that could be construed as a potential conflict of interest.

## References

[B1] Abdi R. D. Gillespie B. E. Vaughn J. Merrill C. Headrick S. I. Ensermu D. B.. (2018). Antimicrobial resistance of *Staphylococcus aureus* isolates from dairy cows and genetic diversity of resistant isolates. Foodborne Pathog. Dis. [Epub ahead of print]. 10.1089/fpd.2017.2362 29394099

[B2] Abe J. Ito Y. Onimaru M. Kohsaka T. Takeda T. (2000). Characterization and distribution of a new enterotoxin-related superantigen produced by *Staphylococcus aureus*. Microbiol. Immunol. 44, 79–88. 10.1111/j.1348-0421.2000.tb01250.x 10803494

[B3] Adhikari R. P. Haudenschild C. Sterba P. M. Sahandi S. Enterlein S. Holtsberg F. W.. (2016). Development of a novel multiplex electrochemiluminescent-based immunoassay for quantification of human serum IgG against 10 *Staphylococcus aureus* toxins. J. Immunol. Methods 430, 33–42. 10.1016/j.jim.2016.01.013 26826278

[B4] Agace W. W. (2008). T-cell recruitment to the intestinal mucosa. Trends Immunol. 29, 514–522. 10.1016/j.it.2008.08.003 18838302

[B5] Aguilar J. L. Varshney A. K. Wang X. Stanford L. Scharff M. Fries B. C. (2014). Detection and measurement of staphylococcal enterotoxin-like K (SEl-K) secretion by *Staphylococcus aureus* clinical isolates. J. Clin. Microbiol. 52, 2536–2543. 10.1128/JCM.00387-14 24808237 PMC4097694

[B6] Alber G. Scheuber P. H. Reck B. Sailer-Kramer B. Hartmann A. Hammer D. K. (1989). Role of substance P in immediate-type skin reactions induced by staphylococcal enterotoxin B in unsensitized monkeys. J. Allergy Clin. Immunol. 84, 880–885. 10.1016/0091-6749(89)90383-7 2480969

[B7] Alibayov B. Zdenkova K. Sykorova H. Demnerova K. (2014). Molecular analysis of *Staphylococcus aureus* pathogenicity islands (SaPI) and their superantigens combination of food samples. J. Microbiol. Methods 107, 197–204. 10.1016/j.mimet.2014.10.014 25447888

[B8] Arad G. Levy R. Nasie I. Hillman D. Rotfogel Z. Barash U.. (2011). Binding of superantigen toxins into the CD28 homodimer interface is essential for induction of cytokine genes that mediate lethal shock. PLoS Biol. 9:e1001149. 10.1371/journal.pbio.1001149 21931534 PMC3172200

[B9] Argudín M. Á. Mendoza M. C. Rodicio M. R. (2010). Food poisoning and *Staphylococcus aureus* enterotoxins. Toxins 2, 1751–1773. 10.3390/toxins2071751 22069659 PMC3153270

[B10] Armstrong-Esther C. A. (1976). Carriage patterns of *Staphylococcus aureus* in a healthy non-hospital population of adults and children. Ann. Hum. Biol. 3, 221–227. 10.1080/03014467600001381 962302

[B11] Asao T. Kumeda Y. Kawai T. Shibata T. Oda H. Haruki K.. (2003). An extensive outbreak of staphylococcal food poisoning due to low-fat milk in Japan: estimation of enterotoxin A in the incriminated milk and powdered skim milk. Epidemiol. Infect. 130, 33–40. 10.1017/S0950268802007951 12613743 PMC2869936

[B12] Baba T. Takeuchi F. Kuroda M. Yuzawa H. Aoki K. Oguchi A.. (2002). Genome and virulence determinants of high virulence community-acquired MRSA. Lancet 359, 1819–1827. 10.1016/S0140-6736(02)08713-5 12044378

[B13] Baroja M. L. Herfst C. A. Kasper K. J. Xu S. X. Gillett D. A. Li J.. (2016). The SaeRS two-component system is a direct and dominant transcriptional activator of toxic shock syndrome toxin 1 in *Staphylococcus aureus*. J. Bacteriol. 198, 2732–2742. 10.1128/JB.00425-16 27457715 PMC5019057

[B14] Bayles K. W. Iandolo J. J. (1989). Genetic and molecular analyses of the gene encoding staphylococcal enterotoxin D. J. Bacteriol. 171, 4799–4806. 10.1128/jb.171.9.4799-4806.1989 2549000 PMC210282

[B15] Becker K. Friedrich A. W. Lubritz G. Weilert M. Peters G. Von Eiff C. (2003). Prevalence of genes encoding pyrogenic toxin superantigens and exfoliative toxins among strains of *Staphylococcus aureus* isolated from blood and nasal specimens. J. Clin. Microbiol. 41, 1434–1439. 10.1128/JCM.41.4.1434-1439.2003 12682126 PMC153929

[B16] Bennett S. D. Walsh K. A. Gould L. H. (2013). Foodborne disease outbreaks caused by *Bacillus cereus, Clostridium perfringens*, and *Staphylococcus aureus*- United States, 1998-2008. Clin. Infect. Dis. 57, 425–433. 10.1093/cid/cit244 23592829 PMC11334977

[B17] Bergdoll M. S. (1983). Enterotoxins, in Staphylococci and Staphylococcal Infections, eds Easton C. S. F. Adlam C. (London: Academic Press), 559–598.

[B18] Bergdoll M. S. (1988). Monkey feeding test for staphylococcal enterotoxin. Methods Enzymol. 165, 324–333. 10.1016/S0076-6879(88)65048-8 3231111

[B19] Bergdoll M. S. Borja C. R. Avena R. M. (1965). Identification of a new enterotoxin as enterotoxin C. J. Bacteriol. 90, 1481–1485. 4954560 10.1128/jb.90.5.1481-1485.1965 PMC315837

[B20] Bergdoll M. S. Borja C. R. Robbins R. N. Weiss K. F. (1971). Identification of enterotoxin E. Infect. Immun. 4, 593–595. 5005309 10.1128/iai.4.5.593-595.1971 PMC416358

[B21] Bergdoll M. S. Crass B. A. Reiser R. F. Robbins R. N. Davis J. P. (1981). A new staphylococcal enterotoxin, enterotoxin F, associated with toxic-shock-syndrome *Staphylococcus aureus* isolates. Lancet 1, 1017–1021. 10.1016/S0140-6736(81)92186-3 6112412

[B22] Bergdoll M. S. Robbins R. N. Weiss K. Borja C. R. Huang Y. Chu F. S. (1973). The staphylococcal enterotoxins: similarities. Contrib. Microbiol. Immunol. 1, 390–396. 4141948

[B23] Betley M. J. Borst D. W. Regassa L. B. (1992). Staphylococcal enterotoxins, toxic shock syndrome toxin and streptococcal pyrogenic exotoxins: a comparative study of their molecular biology. Chem. Immunol. 55, 1–35. 1418613

[B24] Blaiotta G. Ercolini D. Pennacchia C. Fusco V. Casaburi A. Pepe O.. (2004). PCR detection of staphylococcal enterotoxin genes in *Staphylococcus* spp. strains isolated from meat and dairy products. Evidence for new variants of *seg* and *sei* in *S. aureus* AB-8802. J. Appl. Microbiol. 97, 719–730. 10.1111/j.1365-2672.2004.02349.x 15357721

[B25] Bohach G. A. Schlievert P. M. (1987). Nucleotide sequence of the staphylococcal enterotoxin C1 gene and relatedness to other pyrogenic toxins. Mol. Gen. Genet. 209, 15–20. 10.1007/BF00329830 2823067

[B26] Boisset S. Geissmann T. Huntzinger E. Fechter P. Bendridi N. Possedko M.. (2007). *Staphylococcus aureus* RNAIII coordinately represses the synthesis of virulence factors and the transcription regulator Rot by an antisense mechanism. Genes Dev. 21, 1353–1366. 10.1101/gad.423507 17545468 PMC1877748

[B27] Borst D. W. Betley M. J. (1994). Promoter analysis of the staphylococcal enterotoxin A gene. J. Biol. Chem. 269, 1883–1888. 8294437

[B28] Bronner S. Monteil H. Prévost G. (2004). Regulation of virulence determinants in *Staphylococcus aureus*: complexity and applications. FEMS Microbiol. Rev. 28, 183–200. 10.1016/j.femsre.2003.09.003 15109784

[B29] Bruno J. G. Kiel J. L. (2002). Use of magnetic beads in selection and detection of biotoxin aptamers by electrochemiluminescence and enzymatic methods. BioTechniques 32, 178–180, 182–183. 11808691 10.2144/02321dd04

[B30] Callahan J. H. Shefcheck K. J. Williams T. L. Musser S. M. (2006). Detection, confirmation, and quantification of staphylococcal enterotoxin B in food matrixes using liquid chromatography - mass spectrometry. Anal. Chem. 78, 1789–1800. 10.1021/ac051292v 16536413

[B31] Cameron D. R. Howden B. P. Peleg A. Y. (2011). The interface between antibiotic resistance and virulence in *Staphylococcus aureus* and its impact upon clinical outcomes. Clin. Infect. Dis. 53, 576–582. 10.1093/cid/cir473 21865195

[B32] Cao R. Zeaki N. Wallin-Carlquist N. Skandamis P. N. Schelin J. Rådström P. (2012). Elevated enterotoxin A expression and formation in *Staphylococcus aureus* and its association with prophage induction. Appl. Environ. Microbiol. 78, 4942–4948. 10.1128/AEM.00803-12 22544256 PMC3416385

[B33] Carding S. R. Egan P. J. (2002). Gammadelta T cells: functional plasticity and heterogeneity. Nat. Rev. Immunol. 2, 336–345. 10.1038/nri797 12033739

[B34] Carlsson R. Fischer H. Sjögren H. O. (1988). Binding of staphylococcal enterotoxin A to accessory cells is a requirement for its ability to activate human T cells. J. Immunol. 140, 2484–2488. 3258609

[B35] Casman E. P. Bennett R. W. Dorsey A. E. Issa J. A. (1967). Identification of a fourth staphylococcal enterotoxin, enterotoxin D. J. Bacteriol. 94, 1875–1882. 4965366 10.1128/jb.94.6.1875-1882.1967 PMC276916

[B36] Chambers H. F. (2001). The changing epidemiology of *Staphylococcus aureus*? Emerg. Infect. Dis. 7, 178–182. 10.3201/eid0702.010204 11294701 PMC2631711

[B37] Chao G. Bao G. Cao Y. Yan W. Wang Y. Zhang X.. (2015). Prevalence and diversity of enterotoxin genes with genetic background of *Staphylococcus aureus* isolates from different origins in China. Int. J. Food Microbiol. 211, 142–147. 10.1016/j.ijfoodmicro.2015.07.018 26210294

[B38] Cheng J. Wang Y. Cao Y. Yan W. Niu X. Zhou L.. (2016). The distribution of 18 enterotoxin and enterotoxin-like genes in *Staphylococcus aureus* strains from different sources in East China. Foodborne Pathog. Dis. 13, 171–176. 10.1089/fpd.2015.1963 27074376

[B39] Cheung A. L. Projan S. J. (1994). Cloning and sequencing of *sarA* of *Staphylococcus aureus*, a gene required for the expression of *agr*. J. Bacteriol. 176, 4168–4172. 10.1128/jb.176.13.4168-4172.1994 8021198 PMC205618

[B40] Cheung A. L. Bayer A. S. Zhang G. Gresham H. Xiong Y. Q. (2004). Regulation of virulence determinants *in vitro* and *in vivo* in *Staphylococcus aureus*. FEMS Immunol. Med. Microbiol. 40, 1–9. 10.1016/S0928-8244(03)00309-2 14734180

[B41] Cheung A. L. Nishina K. A. Trotonda M. P. Tamber S. (2008). The SarA protein family of *Staphylococcus aureus*. Int. J. Biochem. Cell Biol. 40, 355–361. 10.1016/j.biocel.2007.10.032 18083623 PMC2274939

[B42] Chiang Y. C. Liao W. W. Fan C. M. Pai W. Y. Chiou C. S. Tsen H. Y. (2008). PCR detection of staphylococcal enterotoxins (SEs) N, O, P, Q, R, U, and survey of SE types in *Staphylococcus aureus* isolates from food-poisoning cases in Taiwan. Int. J. Food Microbiol. 121, 66–73. 10.1016/j.ijfoodmicro.2007.10.005 18068843

[B43] Cho H. Jeong D. W. Liu Q. Yeo W. S. Vogl T. Skaar E. P.. (2015). Calprotectin increases the activity of the SaeRS two component system and murine mortality during *Staphylococcus aureus* infections. PLoS Pathog. 11:e1005026. 10.1371/journal.ppat.1005026 26147796 PMC4492782

[B44] Choi Y. W. Herman A. Digiusto D. Wade T. Marrack P. Kappler J. (1990). Residues of the variable region of the T-cell-receptor beta-chain that interact with *S. aureus* toxin superantigens. Nature 346, 471–473. 10.1038/346471a0 2377208

[B45] Collery M. M. Smyth D. S. Tumilty J. J. Twohig J. M. Smyth C. J. (2009). Associations between enterotoxin gene cluster types *egc1, egc2* and *egc3, agr* types, enterotoxin and enterotoxin-like gene profiles, and molecular typing characteristics of human nasal carriage and animal isolates of *Staphylococcus aureus*. J. Med. Microbiol. 58, 13–25. 10.1099/jmm.0.005215-0 19074649

[B46] Compagnone-Post P. Malyankar U. Khan S. A. (1991). Role of host factors in the regulation of the enterotoxin B gene. J. Bacteriol. 173, 1827–1830. 10.1128/jb.173.5.1827-1830.1991 1999394 PMC207339

[B47] Couch J. L. Betley M. J. (1989). Nucleotide sequence of the type C3 staphylococcal enterotoxin gene suggests that intergenic recombination causes antigenic variation. J. Bacteriol. 171, 4507–4510. 10.1128/jb.171.8.4507-4510.1989 2473979 PMC210234

[B48] Cuny C. Wieler L. H. Witte W. (2015). Livestock-associated MRSA: the impact on humans. Antibiotics 4, 521–543. 10.3390/antibiotics4040521 27025639 PMC4790311

[B49] Czop J. K. Bergdoll M. S. (1974). Staphylococcal enterotoxin synthesis during the exponential, transitional, and stationary growth phases. Infect. Immun. 9, 229–235. 4205941 10.1128/iai.9.2.229-235.1974 PMC414791

[B50] Dack G. M. (1937). Staphylococci in relation to food poisoning. Am. J. Public Health Nations. Health. 27, 440–443. 10.2105/AJPH.27.5.440 18014617 PMC1563174

[B51] Danielsen E. M. Hansen G. H. Karlsdóttir E. (2013). *Staphylococcus aureus* enterotoxins A- and B: binding to the enterocyte brush border and uptake by perturbation of the apical endocytic membrane traffic. Histochem. Cell Biol. 139, 513–524. 10.1007/s00418-012-1055-8 23180309

[B52] Deringer J. R. Ely R. J. Monday S. R. Stauffacher C. V. Bohach G. A. (1997). Vbeta-dependent stimulation of bovine and human T cells by host-specific staphylococcal enterotoxins. Infect. Immun. 65, 4048–4054. 9317006 10.1128/iai.65.10.4048-4054.1997 PMC175582

[B53] Derzelle S. Dilasser F. Duquenne M. Deperrois V. (2009). Differential temporal expression of the staphylococcal enterotoxins genes during cell growth. Food Microbiol. 26, 896–904. 10.1016/j.fm.2009.06.007 19835778

[B54] Dinges M. M. Orwin P. M. Schlievert P. M. (2000). Exotoxins of *Staphylococcus aureus*. Clin. Microbiol. Rev. 13, 16–34. 10.1128/CMR.13.1.16-34.2000 10627489 PMC88931

[B55] Do Carmo L. S. Cummings C. Linardi V. R. Dias R. S. De Souza J. M. De Sena M. J.. (2004). A case study of a massive staphylococcal food poisoning incident. Foodborne Pathog. Dis. 1, 241–246. 10.1089/fpd.2004.1.241 15992286

[B56] Doyle M. E. (2015). Multidrug-resistant pathogens in the food supply. Foodborne Pathog. Dis. 12, 261–279. 10.1089/fpd.2014.1865 25621383

[B57] Dusseaux M. Martin E. Serriari N. Péguillet I. Premel V. Louis D.. (2011). Human MAIT cells are xenobiotic-resistant, tissue-targeted, CD161hi IL-17-secreting T cells. Blood 117, 1250–1259. 10.1182/blood-2010-08-303339 21084709

[B58] Edwards L. A. O'neill C. Furman M. A. Hicks S. Torrente F. Pérez-Machado M.. (2012). Enterotoxin-producing staphylococci cause intestinal inflammation by a combination of direct epithelial cytopathy and superantigen-mediated T-cell activation. Inflamm. Bowel Dis. 18, 624–640. 10.1002/ibd.21852 21887731

[B59] Edwards V. M. Deringer J. R. Callantine S. D. Deobald C. F. Berger P. H. Kapur V.. (1997). Characterization of the canine type C enterotoxin produced by *Staphylococcus intermedius* pyoderma isolates. Infect. Immun. 65, 2346–2352. 9169773 10.1128/iai.65.6.2346-2352.1997 PMC175325

[B60] Edwin C. Kass E. H. (1989). Identification of functional antigenic segments of toxic shock syndrome toxin 1 by differential immunoreactivity and by differential mitogenic responses of human peripheral blood mononuclear cells, using active toxin fragments. Infect. Immun. 57, 2230–2236. 2731989 10.1128/iai.57.7.2230-2236.1989 PMC313865

[B61] Ercoli L. Gallina S. Nia Y. Auvray F. Primavilla S. Guidi F.. (2017). Investigation of a staphylococcal food poisoning outbreak from a Chantilly cream dessert, in Umbria (Italy). Foodborne Pathog. Dis. 14, 407–413. 10.1089/fpd.2016.2267 28402712 PMC5512467

[B62] Evenson M. L. Hinds M. W. Bernstein R. S. Bergdoll M. S. (1988). Estimation of human dose of staphylococcal enterotoxin A from a large outbreak of staphylococcal food poisoning involving chocolate milk. Int. J. Food Microbiol. 7, 311–316. 10.1016/0168-1605(88)90057-8 3275329

[B63] Fluit A. C. (2012). Livestock-associated *Staphylococcus aureus*. Clin. Microbiol. Infect. 18, 735–744. 10.1111/j.1469-0691.2012.03846.x 22512702

[B64] Fraser J. D. Proft T. (2008). The bacterial superantigen and superantigen-like proteins. Immunol. Rev. 225, 226–243. 10.1111/j.1600-065X.2008.00681.x 18837785

[B65] Fulton F. (1943). Staphylococcal enterotoxin - With special reference to the kitten test. Br. J. Exp. Pathol. 24, 65–72.

[B66] Gagnaire J. Verhoeven P. O. Grattard F. Rigaill J. Lucht F. Pozzetto B.. (2017). Epidemiology and clinical relevance of *Staphylococcus aureus* intestinal carriage: a systematic review and meta-analysis. Expert Rev. Anti Infect. Ther. 15, 767–785. 10.1080/14787210.2017.1358611 28726558

[B67] Gaskill M. E. Khan S. A. (1988). Regulation of the enterotoxin B gene in *Staphylococcus aureus*. J. Biol. Chem. 263, 6276–6280. 3360783

[B68] Ge B. Mukherjee S. Hsu C. H. Davis J. A. Tran T. T. T. Yang Q.. (2017). MRSA and multidrug-resistant *Staphylococcus aureus* in U.S. retail meats, 2010-2011. Food Microbiol. 62, 289–297. 10.1016/j.fm.2016.10.029 27889161

[B69] Geiger T. Goerke C. Mainiero M. Kraus D. Wolz C. (2008). The virulence regulator Sae of *Staphylococcus aureus*: promoter activities and response to phagocytosis-related signals. J. Bacteriol. 190, 3419–3428. 10.1128/JB.01927-07 18344360 PMC2395011

[B70] Geisinger E. Adhikari R. P. Jin R. Ross H. F. Novick R. P. (2006). Inhibition of *rot* translation by RNAIII, a key feature of *agr* function. Mol. Microbiol. 61, 1038–1048. 10.1111/j.1365-2958.2006.05292.x 16879652

[B71] Gkogka E. Reij M. W. Havelaar A. H. Zwietering M. H. Gorris L. G. (2011). Risk-based estimate of effect of foodborne diseases on public health, Greece. Emerg. Infect. Dis. 17, 1581–1590. 10.3201/eid1709.101766 21888782 PMC3322063

[B72] Goerke C. Pantucek R. Holtfreter S. Schulte B. Zink M. Grumann D.. (2009). Diversity of prophages in dominant *Staphylococcus aureus* clonal lineages. J. Bacteriol. 191, 3462–3468. 10.1128/JB.01804-08 19329640 PMC2681900

[B73] Goldman E. R. Anderson G. P. Tran P. T. Mattoussi H. Charles P. T. Mauro J. M. (2002). Conjugation of luminescent quantum dots with antibodies using an engineered adaptor protein to provide new reagents for fluoroimmunoassays. Anal. Chem. 74, 841–847. 10.1021/ac010662m 11866065

[B74] Grumann D. Scharf S. S. Holtfreter S. Kohler C. Steil L. Engelmann S.. (2008). Immune cell activation by enterotoxin gene cluster (*egc*)-encoded and non-*egc* superantigens from *Staphylococcus aureus*. J. Immunol. 181, 5054–5061. 10.4049/jimmunol.181.7.5054 18802109

[B75] Guinane C. M. Ben Zakour N. L. Tormo-Mas M. A. Weinert L. A. Lowder B. V. Cartwright R. A.. (2010). Evolutionary genomics of *Staphylococcus aureus* reveals insights into the origin and molecular basis of ruminant host adaptation. Genome Biol. Evol. 2, 454–466. 10.1093/gbe/evq031 20624747 PMC2997551

[B76] Gupta G. Bhaskar A. S. Tripathi B. K. Pandey P. Boopathi M. Rao P. V.. (2011). Supersensitive detection of T-2 toxin by the *in situ* synthesized pi-conjugated molecularly imprinted nanopatterns. An *in situ* investigation by surface plasmon resonance combined with electrochemistry. Biosens. Bioelectron. 26, 2534–2540. 10.1016/j.bios.2010.10.050 21111601

[B77] Haag A. F. Bagnoli F. (2016). The role of two-component signal transduction systems in *Staphylococcus aureus* virulence regulation. Curr Top Microbiol. Immunol. 409, 145–198. 10.1007/82_2015_5019 26728068

[B78] Hait J. Tallent S. Melka D. Keys C. Bennett R. (2014). Prevalence of enterotoxins and toxin gene profiles of *Staphylococcus aureus* isolates recovered from a bakery involved in a second staphylococcal food poisoning occurrence. J. Appl. Microbiol. 117, 866–875. 10.1111/jam.12571 24917203

[B79] Hamad A. R. Marrack P. Kappler J. W. (1997). Transcytosis of staphylococcal superantigen toxins. J. Exp. Med. 185, 1447–1454. 10.1084/jem.185.8.1447 9126925 PMC2196287

[B80] Harteveld J. L. Nieuwenhuizen M. S. Wils E. R. (1997). Detection of staphylococcal enterotoxin B employing a piezoelectric crystal immunosensor. Biosens. Bioelectron. 12, 661–667. 10.1016/S0956-5663(97)00024-9 9366022

[B81] Hayworth J. L. Mazzuca D. M. Maleki Vareki S. Welch I. Mccormick J. K. Haeryfar S. M. (2012). CD1d-independent activation of mouse and human iNKT cells by bacterial superantigens. Immunol. Cell Biol. 90, 699–709. 10.1038/icb.2011.90 22041925

[B82] Heinrichs J. H. Bayer M. G. Cheung A. L. (1996). Characterization of the *sar* locus and its interaction with *agr* in *Staphylococcus aureus*. J. Bacteriol. 178, 418–423. 10.1128/jb.178.2.418-423.1996 8550461 PMC177673

[B83] Hennekinne J. A. De Buyser M. L. Dragacci S. (2012). *Staphylococcus aureus* and its food poisoning toxins: characterization and outbreak investigation. FEMS Microbiol. Rev. 36, 815–836. 10.1111/j.1574-6976.2011.00311.x 22091892

[B84] Hirose S. Ono H. K. Omoe K. Hu D. L. Asano K. Yamamoto Y.. (2016). Goblet cells are involved in translocation of staphylococcal enterotoxin A in the intestinal tissue of house musk shrew (*Suncus murinus*). J. Appl. Microbiol. 120, 781–789. 10.1111/jam.13029 26669704

[B85] Ho J. Boost M. O'donoghue M. (2015). Prevalence of enterotoxin genes in *Staphylococcus aureus* colonising food handlers: does nasal carriage status matter? Eur. J. Clin. Microbiol. Infect. Dis. 34, 2177–2181. 10.1007/s10096-015-2465-z 26306787

[B86] Hoffman M. Tremaine M. Mansfield J. Betley M. (1996). Biochemical and mutational analysis of the histidine residues of staphylococcal enterotoxin A. Infect. Immun. 64, 885–890. 8641796 10.1128/iai.64.3.885-890.1996 PMC173852

[B87] Holtfreter S. Bauer K. Thomas D. Feig C. Lorenz V. Roschack K. (2004). *egc*-encoded superantigens from *Staphylococcus aureus* are neutralized by human sera much less efficiently than are classical staphylococcal enterotoxins or toxic shock syndrome toxin. Infect. Immun. 72, 4061–4071. 10.1128/IAI.72.7.4061-4071.2004 15213151 PMC427458

[B88] Holtfreter S. Grumann D. Schmudde M. Nguyen H. T. Eichler P. Strommenger B.. (2007). Clonal distribution of superantigen genes in clinical *Staphylococcus aureus* isolates. J. Clin. Microbiol. 45, 2669–2680. 10.1128/JCM.00204-07 17537946 PMC1951235

[B89] Horn C. C. Kimball B. A. Wang H. Kaus J. Dienel S. Nagy A.. (2013). Why can't rodents vomit? A comparative behavioral, anatomical, and physiological study. PLoS ONE 8:e60537. 10.1371/journal.pone.0060537 23593236 PMC3622671

[B90] Hovde C. J. Marr J. C. Hoffmann M. L. Hackett S. P. Chi Y. I. Crum K. K.. (1994). Investigation of the role of the disulphide bond in the activity and structure of staphylococcal enterotoxin C1. Mol. Microbiol. 13, 897–909. 10.1111/j.1365-2958.1994.tb00481.x 7815947

[B91] Hu D. L. Nakane A. (2014). Mechanisms of staphylococcal enterotoxin-induced emesis. Eur. J. Pharmacol. 722, 95–107. 10.1016/j.ejphar.2013.08.050 24184671

[B92] Hu D. L. Omoe K. Shimoda Y. Nakane A. Shinagawa K. (2003). Induction of emetic response to staphylococcal enterotoxins in the house musk shrew (*Suncus murinus*). Infect. Immun. 71, 567–570. 10.1128/IAI.71.1.567-570.2003 12496213 PMC143409

[B93] Hu D. L. Omoe K. Shimura H. Ono K. Sugii S. Shinagawa K. (1999). Emesis in the shrew mouse (*Suncus murinus*) induced by peroral and intraperitoneal administration of staphylococcal enterotoxin A. J. Food Prot. 62, 1350–1353. 10.4315/0362-028X-62.11.1350 10571329

[B94] Hu D. L. Ono H. K. Isayama S. Okada R. Okamura M. Lei L. C.. (2017). Biological characteristics of staphylococcal enterotoxin Q and its potential risk for food poisoning. J. Appl. Microbiol. 122, 1672–1679. 10.1111/jam.13462 28375567

[B95] Huijsdens X. W. Van Dijke B. J. Spalburg E. Van Santen-Verheuvel M. G. Heck M. E. Pluister G. N.. (2006). Community-acquired MRSA and pig-farming. Ann. Clin. Microbiol. Antimicrob. 5:26. 10.1186/1476-0711-5-26 17096847 PMC1654169

[B96] Humber J. Y. Denny C. B. Bohrer C. W. (1975). Influence of pH on the heat inactivation of staphylococcal enterotoxin A as determined by monkey feeding and serological assay. Appl. Microbiol. 30, 755–758. 950 10.1128/am.30.5.755-758.1975 PMC187267

[B97] Hussain M. A. Dawson C. O. (2013). Economic impact of food safety outbreaks on food businesses. Foods 2, 585–589. 10.3390/foods2040585 28239140 PMC5302274

[B98] Huvenne W. Hellings P. W. Bachert C. (2013). Role of staphylococcal superantigens in airway disease. Int. Arch. Allergy Immunol. 161, 304–314. 10.1159/000350329 23689556

[B99] Igarashi H. (1972). Staphylococcal enterotoxin D. Immunological identification with purified toxin. Jpn. J. Microbiol. 16, 483–491. 10.1111/j.1348-0421.1972.tb00688.x 4632983

[B100] Ikeda T. Tamate N. Yamaguchi K. Makino S. (2005). Mass outbreak of food poisoning disease caused by small amounts of staphylococcal enterotoxins A and H. Appl. Environ. Microbiol. 71, 2793–2795. 10.1128/AEM.71.5.2793-2795.2005 15870376 PMC1087516

[B101] Jarraud S. Peyrat M. A. Lim A. Tristan A. Bes M. Mougel C.. (2001). *egc*, a highly prevalent operon of enterotoxin gene, forms a putative nursery of superantigens in *Staphylococcus aureus*. J. Immunol. 166, 669–677. 10.4049/jimmunol.166.1.669 11123352

[B102] Jett M. Brinkley W. Neill R. Gemski P. Hunt R. (1990). *Staphylococcus aureus* enterotoxin B challenge of monkeys: correlation of plasma levels of arachidonic acid cascade products with occurrence of illness. Infect. Immun. 58, 3494–3499. 2172165 10.1128/iai.58.11.3494-3499.1990 PMC313688

[B103] Ji G. Beavis R. C. Novick R. P. (1995). Cell density control of staphylococcal virulence mediated by an octapeptide pheromone. Proc. Natl. Acad. Sci. U.S.A. 92, 12055–12059. 10.1073/pnas.92.26.12055 8618843 PMC40295

[B104] Ji G. Beavis R. Novick R. P. (1997). Bacterial interference caused by autoinducing peptide variants. Science 276, 2027–2030. 10.1126/science.276.5321.2027 9197262

[B105] Johler S. Giannini P. Jermini M. Hummerjohann J. Baumgartner A. Stephan R. (2015). Further evidence for staphylococcal food poisoning outbreaks caused by *egc*-encoded enterotoxins. Toxins 7, 997–1004. 10.3390/toxins7030997 25802973 PMC4379538

[B106] Johler S. Sihto H. M. Macori G. Stephan R. (2016). Sequence variability in staphylococcal enterotoxin genes *seb, sec*, and *sed*. Toxins. 8:169. 10.3390/toxins8060169 27258311 PMC4926136

[B107] Jørgensen H. J. Mathisen T. Løvseth A. Omoe K. Qvale K. S. Loncarevic S. (2005). An outbreak of staphylococcal food poisoning caused by enterotoxin H in mashed potato made with raw milk. FEMS Microbiol. Lett. 252, 267–272. 10.1016/j.femsle.2005.09.005 16213675

[B108] Kappler J. Kotzin B. Herron L. Gelfand E. W. Bigler R. D. Boylston A.. (1989). V beta-specific stimulation of human T cells by staphylococcal toxins. Science 244, 811–813. 10.1126/science.2524876 2524876

[B109] Kavanaugh J. S. Thoendel M. Horswill A. R. (2007). A role for type I signal peptidase in *Staphylococcus aureus* quorum sensing. Mol. Microbiol. 65, 780–798. 10.1111/j.1365-2958.2007.05830.x 17608791

[B110] Kérouanton A. Hennekinne J. A. Letertre C. Petit L. Chesneau O. Brisabois A.. (2007). Characterization of *Staphylococcus aureus* strains associated with food poisoning outbreaks in France. Int. J. Food Microbiol. 115, 369–375. 10.1016/j.ijfoodmicro.2006.10.050 17306397

[B111] Kientz C. E. Hulst A. G. Wils E. R. (1997). Determination of staphylococcal enterotoxin B by on-line (micro) liquid chromatography-electrospray mass spectrometry. J. Chromatogr. A. 757, 51–64. 10.1016/S0021-9673(96)00661-9 9025260

[B112] Kijek T. M. Rossi C. A. Moss D. Parker R. W. Henchal E. A. (2000). Rapid and sensitive immunomagnetic-electrochemiluminescent detection of staphyloccocal enterotoxin B. J. Immunol. Methods. 236, 9–17. 10.1016/S0022-1759(99)00234-3 10699575

[B113] Kinkel T. L. Roux C. M. Dunman P. M. Fang F. C. (2013). The *Staphylococcus aureus* SrrAB two-component system promotes resistance to nitrosative stress and hypoxia. mBio. 4:e00696-13. 10.1128/mBio.00696-13 24222487 PMC3892780

[B114] Kirk M. Ford L. Glass K. Hall G. (2014). Foodborne illness, Australia, circa 2000 and circa 2010. Emerging Infect. Dis. 20, 1857–1864. 10.3201/eid2011.131315 25340705 PMC4214288

[B115] Kocandrle V. Houttuin E. Prohaska J. V. (1966). Acute hemodynamic and gastrointestinal changes produced by staphylococcal exotoxin and enterotoxin in dogs. J. Surg. Res. 6, 50–57. 10.1016/S0022-4804(66)80070-7 5903206

[B116] Koenig R. L. Ray J. L. Maleki S. J. Smeltzer M. S. Hurlburt B. K. (2004). *Staphylococcus aureus* AgrA binding to the RNAIII-*agr* regulatory region. J. Bacteriol. 186, 7549–7555. 10.1128/JB.186.22.7549-7555.2004 15516566 PMC524880

[B117] Kohler P. L. Greenwood S. D. Nookala S. Kotb M. Kranz D. M. Schlievert P. M. (2012). *Staphylococcus aureus* isolates encode variant staphylococcal enterotoxin B proteins that are diverse in superantigenicity and lethality. PLoS ONE 7:e41157. 10.1371/journal.pone.0041157 22815951 PMC3397982

[B118] Kozono H. Parker D. White J. Marrack P. Kappler J. (1995). Multiple binding sites for bacterial superantigens on soluble class II MHC molecules. Immunity 3, 187–196. 10.1016/1074-7613(95)90088-8 7648392

[B119] Krakauer T. (2013). Update on staphylococcal superantigen-induced signaling pathways and therapeutic interventions. Toxins 5, 1629–1654. 10.3390/toxins5091629 24064719 PMC3798877

[B120] Krakauer T. Pradhan K. Stiles B. G. (2016). Staphylococcal superantigens spark host-mediated danger signals. Front. Immunol. 7:23. 10.3389/fimmu.2016.00023 26870039 PMC4735405

[B121] Kullik I. Giachino P. (1997). The alternative sigma factor sigmaB in *Staphylococcus aureus*: regulation of the sigB operon in response to growth phase and heat shock. Arch. Microbiol. 167, 151–159. 10.1007/s002030050428 9042755

[B122] Kullik I. Giachino P. Fuchs T. (1998). Deletion of the alternative sigma factor sigmaB in *Staphylococcus aureus* reveals its function as a global regulator of virulence genes. J. Bacteriol. 180, 4814–4820. 9733682 10.1128/jb.180.18.4814-4820.1998 PMC107504

[B123] Kuroda H. Kuroda M. Cui L. Hiramatsu K. (2007). Subinhibitory concentrations of beta-lactam induce haemolytic activity in *Staphylococcus aureus* through the SaeRS two-component system. FEMS Microbiol. Lett. 268, 98–105. 10.1111/j.1574-6968.2006.00568.x 17263851

[B124] Kusch K. Hanke K. Holtfreter S. Schmudde M. Kohler C. Erck C.. (2011). The influence of SaeRS and sigma(B) on the expression of superantigens in different *Staphylococcus aureus* isolates. Int. J. Med. Microbiol. 301, 488–499. 10.1016/j.ijmm.2011.01.003 21470910

[B125] Langley R. J. Ting Y. T. Clow F. Young P. G. Radcliff F. J. Choi J. M.. (2017). Staphylococcal enterotoxin-like X (SElX) is a unique superantigen with functional features of two major families of staphylococcal virulence factors. PLoS Pathog. 13:e1006549. 10.1371/journal.ppat.1006549 28880913 PMC5589262

[B126] Langley R. Patel D. Jackson N. Clow F. Fraser J. D. (2010). Staphylococcal superantigen super-domains in immune evasion. Crit. Rev. Immunol. 30, 149–165. 10.1615/CritRevImmunol.v30.i2.40 20370627

[B127] Lappin E. Ferguson A. J. (2009). Gram-positive toxic shock syndromes. Lancet Infect. Dis. 9, 281–290. 10.1016/S1473-3099(09)70066-0 19393958

[B128] Larkin E. A. Carman R. J. Krakauer T. Stiles B. G. (2009). *Staphylococcus aureus*: the toxic presence of a pathogen extraordinaire. Curr. Med. Chem. 16, 4003–4019. 10.2174/092986709789352321 19747126

[B129] Lauderdale K. J. Boles B. R. Cheung A. L. Horswill A. R. (2009). Interconnections between Sigma B, *agr*, and proteolytic activity in *Staphylococcus aureus* biofilm maturation. Infect. Immun. 77, 1623–1635. 10.1128/IAI.01036-08 19188357 PMC2663138

[B130] Le Loir Y. Baron F. Gautier M. (2003). *Staphylococcus aureus* and food poisoning. Genet. Mol. Res. 2, 63–76. 12917803

[B131] Leder L. Llera A. Lavoie P. M. Lebedeva M. I. Li H. Sékaly R. P.. (1998). A mutational analysis of the binding of staphylococcal enterotoxins B and C3 to the T cell receptor beta chain and major histocompatibility complex class II. J. Exp. Med. 187, 823–833. 10.1084/jem.187.6.823 9500785 PMC2212189

[B132] Lee J. Park N. Park J. Y. Kaplan B. L. F. Pruett S. B. Park J. W.. (2017). Induction of immunosuppressive CD8(+)CD25(+)FOXP3(+) regulatory T cells by suboptimal stimulation with staphylococcal enterotoxin C1. J. Immunol. 200, 669–680. 10.4049/jimmunol.1602109 29237775 PMC5757107

[B133] Letertre C. Perelle S. Dilasser F. Fach P. (2003a). Detection and genotyping by real-time PCR of the staphylococcal enterotoxin genes *sea* to *sej*. Mol. Cell. Probes. 17, 139–147. 10.1016/S0890-8508(03)00045-8 12944115

[B134] Letertre C. Perelle S. Dilasser F. Fach P. (2003b). Identification of a new putative enterotoxin SEU encoded by the *egc* cluster of *Staphylococcus aureus*. J. Appl. Microbiol. 95, 38–43. 10.1046/j.1365-2672.2003.01957.x 12807452

[B135] Levy R. Rotfogel Z. Hillman D. Popugailo A. Arad G. Supper E.. (2016). Superantigens hyperinduce inflammatory cytokines by enhancing the B7-2/CD28 costimulatory receptor interaction. Proc. Natl. Acad. Sci. U.S.A. 113, E6437–E6446. 10.1073/pnas.1603321113 27708164 PMC5081635

[B136] Lewis H. C. Mølbak K. Reese C. Aarestrup F. M. Selchau M. Sørum M.. (2008). Pigs as source of methicillin-resistant *Staphylococcus aureus* CC398 infections in humans, Denmark. Emerg. Infect. Dis. 14, 1383–1389. 10.3201/eid1409.071576 18760004 PMC2603104

[B137] Li D. Cheung A. (2008). Repression of *hla* by *rot* is dependent on *sae* in *Staphylococcus aureus*. Infect. Immun. 76, 1068–1075. 10.1128/IAI.01069-07 18174341 PMC2258807

[B138] Li J. Yang J. Lu Y. W. Wu S. Wang M. R. Zhu J. M. (2015). Possible role of staphylococcal enterotoxin B in the pathogenesis of autoimmune diseases. Viral Immunol. 28, 354–359. 10.1089/vim.2015.0017 26086678

[B139] Liang M. Zhang T. Liu X. Fan Y. Xia S. Xiang Y.. (2015). Development of an indirect competitive enzyme-linked immunosorbent assay based on the multiepitope peptide for the synchronous detection of staphylococcal enterotoxin A and G proteins in milk. J. Food Prot. 78, 362–369. 10.4315/0362-028X.JFP-14-323 25710152

[B140] Lina G. Bohach G. A. Nair S. P. Hiramatsu K. Jouvin-Marche E. Mariuzza R.. (2004). Standard nomenclature for the superantigens expressed by *Staphylococcus*. J. Infect. Dis. 189, 2334–2336. 10.1086/420852 15181583

[B141] Lina G. Jarraud S. Ji G. Greenland T. Pedraza A. Etienne J.. (1998). Transmembrane topology and histidine protein kinase activity of AgrC, the *agr* signal receptor in *Staphylococcus aureus*. Mol. Microbiol. 28, 655–662. 10.1046/j.1365-2958.1998.00830.x 9632266

[B142] Lis E. Korzekwa K. Bystron J. Zarczynska A. Dabrowska A. Molenda J.. (2009). Enterotoxin gene content in *Staphylococcus aureus* from the human intestinal tract. FEMS Microbiol. Lett. 296, 72–77. 10.1111/j.1574-6968.2009.01622.x 19459969

[B143] Lis E. Podkowik M. Bystron J. Stefaniak T. Bania J. (2012). Temporal expression of staphylococcal enterotoxin H in comparison with accessory gene regulator-dependent and -independent enterotoxins. J. Food Prot. 75, 238–244. 10.4315/0362-028X.JFP-11-336 22289583

[B144] Lotfi-Emran S. Ward B. R. Le Q. T. Pozez A. L. Manjili M. H. Woodfolk J. A.. (2017). Human mast cells present antigen to autologous CD4(+) T cells. J. Allergy Clin. Immunol. 141, 311.e10–321.e10. 10.1016/j.jaci.2017.02.048 28624612

[B145] Lowder B. V. Guinane C. M. Ben Zakour N. L. Weinert L. A. Conway-Morris A. Cartwright R. A.. (2009). Recent human-to-poultry host jump, adaptation, and pandemic spread of *Staphylococcus aureus*. Proc. Natl. Acad. Sci. U.S.A. 106, 19545–19550. 10.1073/pnas.0909285106 19884497 PMC2780746

[B146] Lowy F. D. (2003). Antimicrobial resistance: the example of *Staphylococcus aureus*. J. Clin. Invest. 111, 1265–1273. 10.1172/JCI18535 12727914 PMC154455

[B147] Luo L. R. Zhang Z. J. Chen L. J. Ma L. F. (2006). Chemiluminescent imaging detection of staphylococcal enterotoxin C-1 in milk and water samples. Food Chem. 97, 355–360. 10.1016/j.foodchem.2005.05.008

[B148] Luu M. Steinhoff U. Visekruna A. (2017). Functional heterogeneity of gut-resident regulatory T cells. Clin. Transl. Immunol. 6:e156. 10.1038/cti.2017.39 28983404 PMC5628268

[B149] Lv G. Xu B. Wei P. Song J. Zhang H. Zhao C.. (2014). Molecular characterization of foodborne-associated *Staphylococcus aureus* strains isolated in Shijiazhuang, China, from 2010 to 2012. Diagn. Microbiol. Infect. Dis. 78, 462–468. 10.1016/j.diagmicrobio.2013.12.006 24582576

[B150] Madsen J. M. (2001). Toxins as weapons of mass destruction. A comparison and contrast with biological-warfare and chemical-warfare agents. Clin. Lab. Med. 21, 593–605. 11577702

[B151] Maeurer M. Zitvogel L. Elder E. Storkus W. J. Lotze M. T. (1995). Human intestinal V delta 1+ T cells obtained from patients with colon cancer respond exclusively to SEB but not to SEA. Nat. Immun. 14, 188–197. 8696008

[B152] Maina E. K. Hu D. L. Tsuji T. Omoe K. Nakane A. (2012). Staphylococcal enterotoxin A has potent superantigenic and emetic activities but not diarrheagenic activity. Int. J. Med. Microbiol. 302, 88–95. 10.1016/j.ijmm.2012.01.003 22424598

[B153] Malachowa N. DeLeo F. R. (2010). Mobile genetic elements of *Staphylococcus aureus*. Cell. Mol. Life Sci. 67, 3057–3071. 10.1007/s00018-010-0389-4 20668911 PMC2929429

[B154] Mangen M. J. Bouwknegt M. Friesema I. H. Haagsma J. A. Kortbeek L. M. Tariq L.. (2015). Cost-of-illness and disease burden of food-related pathogens in the Netherlands, 2011. Int. J. Food Microbiol. 196, 84–93. 10.1016/j.ijfoodmicro.2014.11.022 25528537

[B155] Marr J. C. Lyon J. D. Roberson J. R. Lupher M. Davis W. C. Bohach G. A. (1993). Characterization of novel type C staphylococcal enterotoxins: biological and evolutionary implications. Infect. Immun. 61, 4254–4262. 8406814 10.1128/iai.61.10.4254-4262.1993 PMC281152

[B156] Marrack P. Kappler J. (1990). The staphylococcal enterotoxins and their relatives. Science 248, 705–711. 10.1126/science.2185544 2185544

[B157] Marrack P. Blackman M. Kushnir E. Kappler J. (1990). The toxicity of staphylococcal enterotoxin B in mice is mediated by T cells. J. Exp. Med. 171, 455–464. 10.1084/jem.171.2.455 2303780 PMC2187711

[B158] Mashruwala A. A. Boyd J. M. (2017). The *Staphylococcus aureus* SrrAB regulatory system modulates hydrogen peroxide resistance factors, which imparts protection to aconitase during aerobic growth. PLoS ONE 12:e0170283. 10.1371/journal.pone.0170283 28099473 PMC5242492

[B159] Matsui S. Terabe M. Mabuchi A. Takahashi M. Saizawa M. Tanaka S.. (1997). A unique response to staphylococcal enterotoxin B by intrahepatic lymphocytes and its relevance to the induction of tolerance in the liver. Scand. J. Immunol. 46, 230–234. 10.1046/j.1365-3083.1997.d01-118.x 9315109

[B160] Mayville P. Ji G. Beavis R. Yang H. Goger M. Novick R. P.. (1999). Structure-activity analysis of synthetic autoinducing thiolactone peptides from *Staphylococcus aureus* responsible for virulence. Proc. Natl. Acad. Sci. U.S.A. 96, 1218–1223. 10.1073/pnas.96.4.1218 9990004 PMC15443

[B161] McLauchlin J. Narayanan G. L. Mithani V. O'neill G. (2000). The detection of enterotoxins and toxic shock syndrome toxin genes in *Staphylococcus aureus* by polymerase chain reaction. J. Food Prot. 63, 479–488. 10.4315/0362-028X-63.4.479 10772213

[B162] Miethke T. Wahl C. Heeg K. Echtenacher B. Krammer P. H. Wagner H. (1992). T cell-mediated lethal shock triggered in mice by the superantigen staphylococcal enterotoxin B: critical role of tumor necrosis factor. J. Exp. Med. 175, 91–98. 10.1084/jem.175.1.91 1730929 PMC2119077

[B163] Monday S. R. Bohach G. A. (2001). Genes encoding staphylococcal enterotoxins G and I are linked and separated by DNA related to other staphylococcal enterotoxins. J. Nat. Toxins. 10, 1–8. 11288724

[B164] Morfeldt E. Taylor D. Von Gabain A. Arvidson S. (1995). Activation of alpha-toxin translation in *Staphylococcus aureus* by the trans-encoded antisense RNA, RNAIII. EMBO J. 14, 4569–4577. 7556100 10.1002/j.1460-2075.1995.tb00136.x PMC394549

[B165] Morita C. T. Li H. Lamphear J. G. Rich R. R. Fraser J. D. Mariuzza R. A.. (2001). Superantigen recognition by gammadelta T cells: SEA recognition site for human Vgamma2 T cell receptors. Immunity 14, 331–344. 10.1016/S1074-7613(01)00113-3 11290341

[B166] Munson S. H. Tremaine M. T. Betley M. J. Welch R. A. (1998). Identification and characterization of staphylococcal enterotoxin types G and I from *Staphylococcus aureus*. Infect. Immun. 66, 3337–3348. 9632603 10.1128/iai.66.7.3337-3348.1998 PMC108350

[B167] Murray R. J. (2005). Recognition and management of *Staphylococcus aureus* toxin-mediated disease. Intern. Med. J. 35(Suppl. 2), S106–S119. 10.1111/j.1444-0903.2005.00984.x 16271055

[B168] Nagaraj S. Ramlal S. Kingston J. Batra H. V. (2016). Development of IgY based sandwich ELISA for the detection of staphylococcal enterotoxin G (SEG), an *egc* toxin. Int. J. Food Microbiol. 237, 136–141. 10.1016/j.ijfoodmicro.2016.08.009 27569376

[B169] Napier R. J. Adams E. J. Gold M. C. Lewinsohn D. M. (2015). The role of mucosal associated invariant T cells in antimicrobial immunity. Front. Immunol. 6:344. 10.3389/fimmu.2015.00344 26217338 PMC4492155

[B170] Nashev D. Toshkova K. Bizeva L. Akineden O. Lämmler C. Zschöck M. (2007). Distribution of enterotoxin genes among carriage- and infection-associated isolates of *Staphylococcus aureus*. Lett. Appl. Microbiol. 45, 681–685. 10.1111/j.1472-765X.2007.02254.x 17944839

[B171] Nedelkov D. Rasooly A. Nelson R. W. (2000). Multitoxin biosensor-mass spectrometry analysis: a new approach for rapid, real-time, sensitive analysis of staphylococcal toxins in food. Int. J. Food Microbiol. 60, 1–13. 10.1016/S0168-1605(00)00328-7 11014517

[B172] Neill R. J. Fanning G. R. Delahoz F. Wolff R. Gemski P. (1990). Oligonucleotide probes for detection and differentiation of *Staphylococcus aureus* strains containing genes for enterotoxins A, B, and C and toxic shock syndrome toxin 1. J. Clin. Microbiol. 28, 1514–1518. 2380378 10.1128/jcm.28.7.1514-1518.1990 PMC267980

[B173] Nemati M. Hermans K. Lipinska U. Denis O. Deplano A. Struelens M.. (2008). Antimicrobial resistance of old and recent *Staphylococcus aureus* isolates from poultry: first detection of livestock-associated methicillin-resistant strain ST398. Antimicrob. Agents Chemother. 52, 3817–3819. 10.1128/AAC.00613-08 18663024 PMC2565892

[B174] Nkouawa A. Sako Y. Nakao M. Nakaya K. Ito A. (2009). Loop-mediated isothermal amplification method for differentiation and rapid detection of *Taenia* species. J. Clin. Microbiol. 47, 168–174. 10.1128/JCM.01573-08 19005142 PMC2620829

[B175] Novick R. P. Jiang D. (2003). The staphylococcal *saeRS* system coordinates environmental signals with *agr* quorum sensing. Microbiology 149, 2709–2717. 10.1099/mic.0.26575-0 14523104

[B176] Novick R. P. Projan S. J. Kornblum J. Ross H. F. Ji G. Kreiswirth B.. (1995). The *agr* P2 operon: an autocatalytic sensory transduction system in *Staphylococcus aureus*. Mol. Gen. Genet. 248, 446–458. 10.1007/BF02191645 7565609

[B177] Nowrouzian F. L. Dauwalder O. Meugnier H. Bes M. Etienne J. Vandenesch F.. (2011). Adhesin and superantigen genes and the capacity of *Staphylococcus aureus* to colonize the infantile gut. J. Infect. Dis. 204, 714–721. 10.1093/infdis/jir388 21844297

[B178] Nowrouzian F. L. Lina G. Hodille E. Lindberg E. Hesselmar B. Saalman R.. (2017). Superantigens and adhesins of infant gut commensal *Staphylococcus aureus* strains and association with subsequent development of atopic eczema. Br. J. Dermatol. 176, 439–445. 10.1111/bjd.15138 27761891

[B179] Omoe K. Hu D. L. Ono H. K. Shimizu S. Takahashi-Omoe H. Nakane A.. (2013). Emetic potentials of newly identified staphylococcal enterotoxin-like toxins. Infect. Immun. 81, 3627–3631. 10.1128/IAI.00550-13 23876808 PMC3811759

[B180] Omoe K. Imanishi K. Hu D. L. Kato H. Fugane Y. Abe Y.. (2005). Characterization of novel staphylococcal enterotoxin-like toxin type P. Infect Immun. 73, 5540–5546. 10.1128/IAI.73.9.5540-5546.2005 16113270 PMC1231067

[B181] Ono H. K. Hirose S. Naito I. Sato'o Y. Asano K. Hu D. L.. (2017). The emetic activity of staphylococcal enterotoxins, SEK, SEL, SEM, SEN and SEO in a small emetic animal model, the house musk shrew. Microbiol. Immunol. 61, 12–16. 10.1111/1348-0421.12460 28042656

[B182] Ono H. K. Nishizawa M. Yamamoto Y. Hu D. L. Nakane A. Shinagawa K.. (2012). Submucosal mast cells in the gastrointestinal tract are a target of staphylococcal enterotoxin type A. FEMS Immunol. Med. Microbiol. 64, 392–402. 10.1111/j.1574-695X.2011.00924.x 22211567

[B183] Ono H. K. Omoe K. Imanishi K. Iwakabe Y. Hu D. L. Kato H.. (2008). Identification and characterization of two novel staphylococcal enterotoxins, types S and T. Infect. Immun. 76, 4999–5005. 10.1128/IAI.00045-08 18710864 PMC2573384

[B184] Ono H. K. Sato'o Y. Narita K. Naito I. Hirose S. Hisatsune J.. (2015). Identification and characterization of a novel staphylococcal emetic toxin. Appl. Environ. Microbiol. 81, 7034–7040. 10.1128/AEM.01873-15 26231643 PMC4579459

[B185] Orwin P. M. Leung D. Y. Donahue H. L. Novick R. P. Schlievert P. M. (2001). Biochemical and biological properties of staphylococcal enterotoxin K. Infect. Immun. 69, 360–366. 10.1128/IAI.69.1.360-366.2001 11119525 PMC97891

[B186] Orwin P. M. Leung D. Y. Tripp T. J. Bohach G. A. Earhart C. A. Ohlendorf D. H.. (2002). Characterization of a novel staphylococcal enterotoxin-like superantigen, a member of the group V subfamily of pyrogenic toxins. Biochemistry 41, 14033–14040. 10.1021/bi025977q 12437361

[B187] Ostyn A. De Buyser M. L. Guillier F. Groult J. Felix B. Salah S.. (2010). First evidence of a food poisoning outbreak due to staphylococcal enterotoxin type E, France, 2009. Euro Surveill. 15:19528 20394711

[B188] Otero A. García M. L. García M. C. Moreno B. Bergdoll M. S. (1990). Production of staphylococcal enterotoxins C1 and C2 and thermonuclease throughout the growth cycle. Appl. Environ. Microbiol. 56, 555–559. 2306093 10.1128/aem.56.2.555-559.1990 PMC183377

[B189] Otto M. (2010). Basis of virulence in community-associated methicillin-resistant *Staphylococcus aureus*. Annu. Rev. Microbiol. 64, 143–162. 10.1146/annurev.micro.112408.134309 20825344

[B190] Otto M. (2012). MRSA virulence and spread. Cell Microbiol. 14, 1513–1521. 10.1111/j.1462-5822.2012.01832.x 22747834 PMC3443268

[B191] Pané-Farré J. Jonas B. Förstner K. Engelmann S. Hecker M. (2006). The sigmaB regulon in *Staphylococcus aureus* and its regulation. Int. J. Med. Microbiol. 296, 237–258. 10.1016/j.ijmm.2005.11.011 16644280

[B192] Park M. S. Kim Y. S. Lee S. H. Kim S. H. Park K. H. Bahk G. J. (2015). Estimating the burden of foodborne disease, South Korea, 2008-2012. Foodborne Pathog. Dis. 12, 207–213. 10.1089/fpd.2014.1858 25622301

[B193] Pastacaldi C. Lewis P. Howarth P. (2011). Staphylococci and staphylococcal superantigens in asthma and rhinitis: a systematic review and meta-analysis. Allergy 66, 549–555. 10.1111/j.1398-9995.2010.02502.x 21087214

[B194] Pekdemir M. E. Ertürkan D. Külah H. Boyaci I. H. Ozgen C. Tamer U. (2012). Ultrasensitive and selective homogeneous sandwich immunoassay detection by Surface Enhanced Raman Scattering (SERS). Analyst 137, 4834–4840. 10.1039/c2an35471c 22943047

[B195] Pinchuk I. V. Beswick E. J. Reyes V. E. (2010). Staphylococcal enterotoxins. Toxins 2, 2177–2197. 10.3390/toxins2082177 22069679 PMC3153290

[B196] Powell N. Macdonald T. T. (2017). Recent advances in gut immunology. Parasite Immunol. 39:e12430. 10.1111/pim.12430 28370104

[B197] Pragman A. A. Ji Y. Schlievert P. M. (2007). Repression of *Staphylococcus aureus* SrrAB using inducible antisense *srrA* alters growth and virulence factor transcript levels. Biochemistry 46, 314–321. 10.1021/bi0603266 17198402

[B198] Pragman A. A. Yarwood J. M. Tripp T. J. Schlievert P. M. (2004). Characterization of virulence factor regulation by SrrAB, a two-component system in *Staphylococcus aureus*. J. Bacteriol. 186, 2430–2438. 10.1128/JB.186.8.2430-2438.2004 15060046 PMC412142

[B199] Price L. B. Stegger M. Hasman H. Aziz M. Larsen J. Andersen P. S.. (2012). *Staphylococcus aureus* CC398: host adaptation and emergence of methicillin resistance in livestock. MBio. 3:e00520-12. 10.1128/mBio.00305-11 22354957 PMC3280451

[B200] Principato M. Qian B. F. (2014). Staphylococcal enterotoxins in the etiopathogenesis of mucosal autoimmunity within the gastrointestinal tract. Toxins 6, 1471–1489. 10.3390/toxins6051471 24776983 PMC4052247

[B201] Queck S. Y. Jameson-Lee M. Villaruz A. E. Bach T. H. Khan B. A. Sturdevant D. E.. (2008). RNAIII-independent target gene control by the *agr* quorum-sensing system: insight into the evolution of virulence regulation in *Staphylococcus aureus*. Mol. Cell. 32, 150–158. 10.1016/j.molcel.2008.08.005 18851841 PMC2575650

[B202] Rasooly L. Rasooly A. (1999). Real time biosensor analysis of staphylococcal enterotoxin A in food. Int. J. Food Microbiol. 49, 119–127. 10.1016/S0168-1605(99)00053-7 10490222

[B203] Read R. B. Jr. Bradshaw J. Pritchard W. L. Black L. A. (1965). Assay of staphylococcal enterotoxin from cheese. J. Dairy Sci. 48, 420–424. 10.3168/jds.S0022-0302(65)88246-7 14282435

[B204] Regassa L. B. Betley M. J. (1993). High sodium chloride concentrations inhibit staphylococcal enterotoxin C gene (*sec*) expression at the level of *sec* mRNA. Infect. Immun. 61, 1581–1585. 8454367 10.1128/iai.61.4.1581-1585.1993 PMC281406

[B205] Regassa L. B. Couch J. L. Betley M. J. (1991). Steady-state staphylococcal enterotoxin type C mRNA is affected by a product of the accessory gene regulator (*agr*) and by glucose. Infect. Immun. 59, 955–962. 1997441 10.1128/iai.59.3.955-962.1991 PMC258352

[B206] Regenthal P. Hansen J. S. Andre I. Lindkvist-Petersson K. (2017). Thermal stability and structural changes in bacterial toxins responsible for food poisoning. PLoS ONE 12:e0172445 10.1371/journal.pone.0172445 28207867 PMC5313198

[B207] Reiser R. F. Robbins R. N. Khoe G. P. Bergdoll M. S. (1983). Purification and some physicochemical properties of toxic-shock toxin. Biochemistry 22, 3907–3912. 10.1021/bi00285a028 6615808

[B208] Reiser R. F. Robbins R. N. Noleto A. L. Khoe G. P. Bergdoll M. S. (1984). Identification, purification, and some physicochemical properties of staphylococcal enterotoxin C3. Infect. Immun. 45, 625–630. 6469349 10.1128/iai.45.3.625-630.1984 PMC263340

[B209] Ridley M. (1959). Perineal carriage of *Staph. aureus*. Br. Med. J. 1, 270–273. 10.1136/bmj.1.5117.270 13618615 PMC1992404

[B210] Rieder S. A. Nagarkatti P. Nagarkatti M. (2011). CD1d-independent activation of invariant natural killer T cells by staphylococcal enterotoxin B through major histocompatibility complex class II/T cell receptor interaction results in acute lung injury. Infect. Immun. 79, 3141–3148. 10.1128/IAI.00177-11 21628519 PMC3147567

[B211] Rogasch K. Rühmling V. Pané-Farré J. Höper D. Weinberg C. Fuchs S.. (2006). Influence of the two-component system SaeRS on global gene expression in two different *Staphylococcus aureus* strains. J. Bacteriol. 188, 7742–7758. 10.1128/JB.00555-06 17079681 PMC1636327

[B212] Sahibzada S. Abraham S. Coombs G. W. Pang S. Hernández-Jover M. Jordan D.. (2017). Transmission of highly virulent community-associated MRSA ST93 and livestock-associated MRSA ST398 between humans and pigs in Australia. Sci. Rep. 7:5273. 10.1038/s41598-017-04789-0 28706213 PMC5509732

[B213] Saïd-Salim B. Dunman P. M. Mcaleese F. M. Macapagal D. Murphy E. Mcnamara P. J.. (2003). Global regulation of *Staphylococcus aureus* genes by Rot. J. Bacteriol. 185, 610–619. 10.1128/JB.185.2.610-619.2003 12511508 PMC145333

[B214] Sakai F. Ihara H. Aoyama K. Igarashi H. Yanahira S. Ohkubo T.. (2008). Characteristics of enterotoxin H-producing *Staphylococcus aureus* isolated from clinical cases and properties of the enterotoxin productivity. J. Food Prot. 71, 1855–1860. 10.4315/0362-028X-71.9.1855 18810869

[B215] Salerno-Goncalves R. Luo D. Fresnay S. Magder L. Darton T. C. Jones C.. (2017). Challenge of humans with wild-type *Salmonella* enterica serovar *Typhi* elicits changes in the activation and homing characteristics of Mucosal-Associated Invariant T Cells. Front. Immunol. 8:398. 10.3389/fimmu.2017.00398 28428786 PMC5382150

[B216] Saline M. Rodstrom K. E. Fischer G. Orekhov V. Y. Karlsson B. G. Lindkvist-Petersson K. (2010). The structure of superantigen complexed with TCR and MHC reveals novel insights into superantigenic T cell activation. Nat. Commun. 1:119. 10.1038/ncomms1117 21081917

[B217] Salomon L. L. Tew R. W. (1968). Assay of staphylococcal enterotoxin B by latex agglutination. Proc. Soc. Exp. Biol. Med. 129, 539–542. 10.3181/00379727-129-33364 5696780

[B218] Sato'o Y. Hisatsune J. Nagasako Y. Ono H. K. Omoe K. Sugai M. (2015). Positive regulation of staphylococcal enterotoxin H by Rot (Repressor of Toxin) protein and its importance in clonal complex 81 subtype 1 lineage-related food poisoning. Appl. Environ. Microbiol. 81, 7782–7790. 10.1128/AEM.01936-15 26341202 PMC4616944

[B219] Sato'o Y. Omoe K. Naito I. Ono H. K. Nakane A. Sugai M.. (2014). Molecular epidemiology and identification of a *Staphylococcus aureus* clone causing food poisoning outbreaks in Japan. J. Clin. Microbiol. 52, 2637–2640. 10.1128/JCM.00661-14 24759723 PMC4097684

[B220] Saunders G. C. Bartlett M. L. (1977). Double-antibody solid-phase enzyme immunoassay for the detection of staphylococcal enterotoxin A. Appl. Environ. Microbiol. 34, 518–522. 337898 10.1128/aem.34.5.518-522.1977 PMC242693

[B221] Scallan E. Hoekstra R. M. Angulo F. J. Tauxe R. V. Widdowson M. A. Roy S. L.. (2011). Foodborne illness acquired in the United States - major pathogens. Emerging Infect. Dis. 17, 7–15. 10.3201/eid1701.P11101 21192848 PMC3375761

[B222] Schantz E. J. Roessler W. G. Wagman J. Spero L. Dunnery D. A. Bergdoll M. S. (1965). Purification of staphylococcal enterotoxin B. Biochemistry 4, 1011–1016. 10.1021/bi00882a005 4953912

[B223] Scheuber P. H. Denzlinger C. Wilker D. Beck G. Keppler D. Hammer D. K. (1987). Staphylococcal enterotoxin B as a nonimmunological mast cell stimulus in primates: the role of endogenous cysteinyl leukotrienes. Int. Arch. Allergy Appl. Immunol. 82, 289–291. 10.1159/000234209 3032802

[B224] Scheuber P. H. Mossmann H. Beck G. Hammer D. K. (1983). Direct skin test in highly sensitized guinea pigs for rapid and sensitive determination of staphylococcal enterotoxin B. Appl. Environ. Microbiol. 46, 1351–1356. 6660875 10.1128/aem.46.6.1351-1356.1983 PMC239575

[B225] Schlievert P. M. Jablonski L. M. Roggiani M. Sadler I. Callantine S. Mitchell D. T.. (2000). Pyrogenic toxin superantigen site specificity in toxic shock syndrome and food poisoning in animals. Infect. Immun. 68, 3630–3634. 10.1128/IAI.68.6.3630-3634.2000 10816521 PMC97652

[B226] Senn L. Clerc O. Zanetti G. Basset P. Prod'hom G. Gordon N. C.. (2016). The stealthy superbug: the role of asymptomatic enteric carriage in maintaining a long-term hospital outbreak of ST228 methicillin-resistant *Staphylococcus aureus*. MBio. 7, e02039–e02015. 10.1128/mBio.02039-15 26787833 PMC4725017

[B227] Sergelidis D. Angelidis A. S. (2017). Methicillin-resistant *Staphylococcus aureus*: a controversial food-borne pathogen. Lett. Appl. Microbiol. 64, 409–418. 10.1111/lam.12735 28304109

[B228] Shaler C. R. Choi J. Rudak P. T. Memarnejadian A. Szabo P. A. Tun-Abraham M. E.. (2017). MAIT cells launch a rapid, robust and distinct hyperinflammatory response to bacterial superantigens and quickly acquire an anergic phenotype that impedes their cognate antimicrobial function: defining a novel mechanism of superantigen-induced immunopathology and immunosuppression. PLoS Biol. 15:e2001930. 10.1371/journal.pbio.2001930 28632753 PMC5478099

[B229] Sheahan D. G. Jervis H. R. Takeuchi A. Sprinz H. (1970). The effect of staphylococcal enterotoxin on the epithelial mucosubstances of the small intestine of rhesus monkeys. Am. J. Pathol. 60, 1–18. 4193441 PMC2032886

[B230] Shen M. Li Y. Zhang L. Dai S. Wang J. Li Y.. (2017). Staphylococcus enterotoxin profile of China isolates and the superantigenicity of some novel enterotoxins. Arch. Microbiol. 199, 723–736. 10.1007/s00203-017-1345-6 28235987

[B231] Shin E. Hong H. Park J. Oh Y. Jung J. Lee Y. (2016). Characterization of *Staphylococcus aureus* faecal isolates associated with food-borne disease in Korea. J. Appl. Microbiol. 121, 277–286. 10.1111/jam.13133 26991816

[B232] Shupp J. W. Jett M. Pontzer C. H. (2002). Identification of a transcytosis epitope on staphylococcal enterotoxins. Infect. Immun. 70, 2178–2186. 10.1128/IAI.70.4.2178-2186.2002 11895985 PMC127880

[B233] Shylaja R. Murali H. S. Batra H. V. Bawa A. S. (2010). A novel multiplex PCR system for the detection of staphylococcal enterotoxin B, *tsst, nuc* and *fem* genes of *Staphylococcus aureus* in food system. J. Food Saf. 30, 443–454. 10.1111/j.1745-4565.2010.00218.x

[B234] Smith T. C. (2015). Livestock-associated *Staphylococcus aureus*: the United States experience. PLoS Pathog. 11:e1004564. 10.1371/journal.ppat.1004564 25654425 PMC4412291

[B235] Song M. Shi C. Xu X. Shi X. (2016). Molecular typing and virulence gene profiles of enterotoxin gene cluster (*egc*)-positive *Staphylococcus aureus* isolates obtained from various food and clinical specimens. Foodborne Pathog. Dis. 13, 592–601. 10.1089/fpd.2016.2162 27792397

[B236] Soykut E. A. Dudak F. C. Boyaci I. H. (2008). Selection of staphylococcal enterotoxin B (SEB)-binding peptide using phage display technology. Biochem. Biophys. Res. Commun. 370, 104–108. 10.1016/j.bbrc.2008.03.065 18359289 PMC7117543

[B237] Spaan A. N. Surewaard B. G. Nijland R. Van Strijp J. A. (2013). Neutrophils versus *Staphylococcus aureus*: a biological tug of war. Annu. Rev. Microbiol. 67, 629–650. 10.1146/annurev-micro-092412-155746 23834243

[B238] Spaulding A. R. Salgado-Pabón W. Kohler P. L. Horswill A. R. Leung D. Y. Schlievert P. M. (2013). Staphylococcal and streptococcal superantigen exotoxins. Clin. Microbiol. Rev. 26, 422–447. 10.1128/CMR.00104-12 23824366 PMC3719495

[B239] Stohl W. Elliott J. E. Linsley P. S. (1994). Human T cell-dependent B cell differentiation induced by staphylococcal superantigens. J. Immunol. 153, 117–127. 7515921

[B240] Su Y. C. Wong A. C. (1995). Identification and purification of a new staphylococcal enterotoxin, H. Appl. Environ. Microbiol. 61, 1438–1443. 7747964 10.1128/aem.61.4.1438-1443.1995 PMC167401

[B241] Su Y. C. Wong A. C. (1996). Detection of staphylococcal enterotoxin H by an enzyme-linked immunosorbent assay. J. Food Prot. 59, 327–330. 10.4315/0362-028X-59.3.327 10463455

[B242] Sugiyama H. Hayama T. (1965). Abdominal viscera as site of emetic action for staphylococcal enterotoxin in the monkey. J. Infect. Dis. 115, 330–336. 10.1093/infdis/115.4.330 4953783

[B243] Suleiman T. S. Karimuribo E. D. Mdegela R. H. (2018). Prevalence of bovine subclinical mastitis and antibiotic susceptibility patterns of major mastitis pathogens isolated in Unguja island of Zanzibar, Tanzania. Trop. Anim. Health Prod. 50, 259–266. 10.1007/s11250-017-1424-3 28980098

[B244] Sullivan R. (1969). Effects of enterotoxin B on intestinal transport *in vitro*. Proc. Soc. Exp. Biol. Med. 131, 1159–1162. 10.3181/00379727-131-34060 5811968

[B245] Sumby P. Waldor M. K. (2003). Transcription of the toxin genes present within the staphylococcal phage phiSa3ms is intimately linked with the phage's life cycle. J. Bacteriol. 185, 6841–6851. 10.1128/JB.185.23.6841-6851.2003 14617648 PMC262704

[B246] Sun S. Yang M. Kostov Y. Rasooly A. (2010). ELISA-LOC: lab-on-a-chip for enzyme-linked immunodetection. Lab Chip. 10, 2093–2100. 10.1039/c003994b 20544092

[B247] Szabo P. A. Rudak P. T. Choi J. Xu S. X. Schaub R. Singh B.. (2017). Invariant natural killer T Cells are pathogenic in the HLA-DR4-transgenic humanized mouse model of toxic shock syndrome and can be targeted to reduce morbidity. J. Infect. Dis. 215, 824–829. 10.1093/infdis/jiw646 28035011

[B248] Taylor S. L. Schlunz L. R. Beery J. T. Cliver D. O. Bergdoll M. S. (1982). Emetic action of staphylococcal enterotoxin A on weanling pigs. Infect. Immun. 36, 1263–1266. 7095851 10.1128/iai.36.3.1263-1266.1982 PMC551469

[B249] Tempelman L. A. King K. D. Anderson G. P. Ligler F. S. (1996). Quantitating staphylococcal enterotoxin B in diverse media using a portable fiber-optic biosensor. Anal. Biochem. 233, 50–57. 10.1006/abio.1996.0006 8789146

[B250] Thomas D. Y. Jarraud S. Lemercier B. Cozon G. Echasserieau K. Etienne J.. (2006). Staphylococcal enterotoxin-like toxins U2 and V, two new staphylococcal superantigens arising from recombination within the enterotoxin gene cluster. Infect. Immun. 74, 4724–4734. 10.1128/IAI.00132-06 16861660 PMC1539601

[B251] Thomas D. Chou S. Dauwalder O. Lina G. (2007). Diversity in *Staphylococcus aureus* enterotoxins. Chem. Immunol. Allergy 93, 24–41. 10.1159/000100856 17369698

[B252] Thomas M. K. Murray R. Flockhart L. Pintar K. Pollari F. Fazil A.. (2013). Estimates of the burden of foodborne illness in Canada for 30 specified pathogens and unspecified agents, circa 2006. Foodborne Pathog. Dis. 10, 639–648. 10.1089/fpd.2012.1389 23659355 PMC3696931

[B253] Tiedemann R. E. Fraser J. D. (1996). Cross-linking of MHC class II molecules by staphylococcal enterotoxin A is essential for antigen-presenting cell and T cell activation. J. Immunol. 157, 3958–3966. 8892628

[B254] Treiner E. Duban L. Bahram S. Radosavljevic M. Wanner V. Tilloy F.. (2003). Selection of evolutionarily conserved mucosal-associated invariant T cells by MR1. Nature 422, 164–169. 10.1038/nature01433 12634786

[B255] Tremaine M. T. Brockman D. K. Betley M. J. (1993). Staphylococcal enterotoxin A gene (*sea*) expression is not affected by the accessory gene regulator (*agr*). Infect. Immun. 61, 356–359. 7678101 10.1128/iai.61.1.356-359.1993 PMC302730

[B256] Tseng C. W. Stewart G. C. (2005). Rot repression of enterotoxin B expression in *Staphylococcus aureus*. J. Bacteriol. 187, 5301–5309. 10.1128/JB.187.15.5301-5309.2005 16030224 PMC1196012

[B257] Tseng C. W. Zhang S. Stewart G. C. (2004). Accessory gene regulator control of staphyloccoccal enterotoxin D gene expression. J. Bacteriol. 186, 1793–1801. 10.1128/JB.186.6.1793-1801.2004 14996810 PMC355899

[B258] Tuffs S. W. James D. B. A. Bestebroer J. Richards A. C. Goncheva M. I. O'shea M.. (2017). The *Staphylococcus aureus* superantigen SElX is a bifunctional toxin that inhibits neutrophil function. PLoS Pathog. 13:e1006461. 10.1371/journal.ppat.1006461 28880920 PMC5589267

[B259] Umeda K. Nakamura H. Yamamoto K. Nishina N. Yasufuku K. Hirai Y.. (2017). Molecular and epidemiological characterization of staphylococcal foodborne outbreak of *Staphylococcus aureus* harboring *seg, sei, sem, sen, seo*, and *selu* genes without production of classical enterotoxins. Int. J. Food Microbiol. 256, 30–35. 10.1016/j.ijfoodmicro.2017.05.023 28582663

[B260] Van Cauteren D. Le Strat Y. Sommen C. Bruyand M. Tourdjman M. Da Silva N. J.. (2017). Estimated annual numbers of foodborne pathogen-associated illnesses, hospitalizations, and deaths, France, 2008-2013. Emerg. Infect. Dis. 23, 1486–1492. 10.3201/eid2309.170081 28820137 PMC5572882

[B261] Van Gessel Y. A. Mani S. Bi S. Hammamieh R. Shupp J. W. Das R.. (2004). Functional piglet model for the clinical syndrome and postmortem findings induced by staphylococcal enterotoxin B. Exp. Biol. Med. 229, 1061–1071. 10.1177/153537020422901011 15522843

[B262] Verhoeven P. O. Gagnaire J. Botelho-Nevers E. Grattard F. Carricajo A. Lucht F.. (2014). Detection and clinical relevance of *Staphylococcus aureus* nasal carriage: an update. Expert Rev. Anti Infect. Ther. 12, 75–89. 10.1586/14787210.2014.859985 24308709

[B263] Viçosa G. N. Le Loir A. Le Loir Y. De Carvalho A. F. Nero L. A. (2013). *egc* characterization of enterotoxigenic *Staphylococcus aureus* isolates obtained from raw milk and cheese. Int. J. Food Microbiol. 165, 227–230. 10.1016/j.ijfoodmicro.2013.05.023 23800734

[B264] Von Eiff C. Becker K. Machka K. Stammer H. Peters G. (2001). Nasal carriage as a source of *Staphylococcus aureus* bacteremia. Study Group. N. Engl. J. Med. 344, 11–16. 10.1056/NEJM200101043440102 11136954

[B265] Wallin-Carlquist N. Cao R. Márta D. Da Silva A. S. Schelin J. Rådström P. (2010). Acetic acid increases the phage-encoded enterotoxin A expression in *Staphylococcus aureus*. BMC Microbiol. 10:147. 10.1186/1471-2180-10-147 20487538 PMC2891721

[B266] Waters C. M. Bassler B. L. (2005). Quorum sensing: cell-to-cell communication in bacteria. Annu. Rev. Cell Dev. Biol. 21, 319–346. 10.1146/annurev.cellbio.21.012704.131001 16212498

[B267] Wattinger L. Stephan R. Layer F. Johler S. (2012). Comparison of *Staphylococcus aureus* isolates associated with food intoxication with isolates from human nasal carriers and human infections. Eur. J. Clin. Microbiol. Infect. Dis. 31, 455–464. 10.1007/s10096-011-1330-y 21761125

[B268] Wertheim H. F. Verveer J. Boelens H. A. Van Belkum A. Verbrugh H. A. Vos M. C. (2005). Effect of mupirocin treatment on nasal, pharyngeal, and perineal carriage of *Staphylococcus aureus* in healthy adults. Antimicrob. Agents Chemother. 49, 1465–1467. 10.1128/AAC.49.4.1465-1467.2005 15793127 PMC1068605

[B269] White J. Herman A. Pullen A. M. Kubo R. Kappler J. W. Marrack P. (1989). The V beta-specific superantigen staphylococcal enterotoxin B: stimulation of mature T cells and clonal deletion in neonatal mice. Cell 56, 27–35. 10.1016/0092-8674(89)90980-X 2521300

[B270] Williams R. E. (1963). Healthy carriage of *Staphylococcus aureus*: its prevalence and importance. Bacteriol. Rev. 27, 56–71. 14000926 10.1128/br.27.1.56-71.1963 PMC441169

[B271] Wilson G. J. Seo K. S. Cartwright R. A. Connelley T. Chuang-Smith O. N. Merriman J. A.. (2011). A novel core genome-encoded superantigen contributes to lethality of community-associated MRSA necrotizing pneumonia. PLoS Pathog. 7:e1002271. 10.1371/journal.ppat.1002271 22022262 PMC3192841

[B272] Wilson I. G. Cooper J. E. Gilmour A. (1991). Detection of enterotoxigenic *Staphylococcus aureus* in dried skimmed milk: use of the polymerase chain reaction for amplification and detection of staphylococcal enterotoxin genes *entB* and *entC1* and the thermonuclease gene nuc. Appl. Environ. Microbiol. 57, 1793–1798. 1872609 10.1128/aem.57.6.1793-1798.1991 PMC183470

[B273] Wright A. Andrews P. L. Titball R. W. (2000). Induction of emetic, pyrexic, and behavioral effects of *Staphylococcus aureus* enterotoxin B in the ferret. Infect. Immun. 68, 2386–2389. 10.1128/IAI.68.4.2386-2389.2000 10722650 PMC97434

[B274] Wu S. de Lencastre H. Tomasz A. (1996). Sigma-B, a putative operon encoding alternate sigma factor of *Staphylococcus aureus* RNA polymerase: molecular cloning and DNA sequencing. J. Bacteriol. 178, 6036–6042. 10.1128/jb.178.20.6036-6042.1996 8830703 PMC178463

[B275] Wu S. Duan N. Gu H. Hao L. Ye H. Gong W.. (2016). A review of the methods for detection of *Staphylococcus aureus* enterotoxins. Toxins 8:176. 10.3390/toxins8070176 27348003 PMC4963824

[B276] Yan X. Wang B. Tao X. Hu Q. Cui Z. Zhang J.. (2012). Characterization of *Staphylococcus aureus* strains associated with food poisoning in Shenzhen, China. Appl. Environ. Microbiol. 78, 6637–6642. 10.1128/AEM.01165-12 22798367 PMC3426705

[B277] Yarwood J. M. Mccormick J. K. Schlievert P. M. (2001). Identification of a novel two-component regulatory system that acts in global regulation of virulence factors of *Staphylococcus aureus*. J. Bacteriol. 183, 1113–1123. 10.1128/JB.183.4.1113-1123.2001 11157922 PMC94983

[B278] Zeaki N. Susilo Y. B. Pregiel A. Rådström P. Schelin J. (2015). Prophage-encoded staphylococcal enterotoxin A: regulation of production in *Staphylococcus aureus* strains representing different *sea* regions. Toxins 7, 5359–5376. 10.3390/toxins7124889 26690218 PMC4690139

[B279] Zhang S. Stewart G. C. (2000). Characterization of the promoter elements for the staphylococcal enterotoxin D gene. J. Bacteriol. 182, 2321–2325. 10.1128/JB.182.8.2321-2325.2000 10735879 PMC111285

[B280] Zhang S. Iandolo J. J. Stewart G. C. (1998). The enterotoxin D plasmid of *Staphylococcus aureus* encodes a second enterotoxin determinant (*sej*). FEMS Microbiol. Lett. 168, 227–233. 10.1111/j.1574-6968.1998.tb13278.x 9835033

[B281] Zhao W. Li Y. Liu W. Ding D. Xu Y. Pan L.. (2016a). Transcytosis, antitumor activity and toxicity of staphylococcal enterotoxin C2 as an oral administration protein drug. Toxins 8:185. 10.3390/toxins8060185 27322320 PMC4926151

[B282] Zhao Y. Zhu A. Tang J. Tang C. Chen J. (2017). Identification and measurement of staphylococcal enterotoxin M from *Staphylococcus aureus* isolate associated with staphylococcal food poisoning. Lett. Appl. Microbiol. 65, 27–34. 10.1111/lam.12751 28444877

[B283] Zhao Y. Zhu A. Tang J. Tang C. Chen J. Liu J. (2016b). Identification and measurement of staphylococcal enterotoxin-like protein I (SEll) secretion from *Staphylococcus aureus* clinical isolate. J. Appl. Microbiol. 121, 539–546. 10.1111/jam.13181 27187155

[B284] Ziebandt A. K. Becher D. Ohlsen K. Hacker J. Hecker M. Engelmann S. (2004). The influence of *agr* and sigmaB in growth phase dependent regulation of virulence factors in *Staphylococcus aureus*. Proteomics 4, 3034–3047. 10.1002/pmic.200400937 15378746

[B285] Ziebandt A. K. Weber H. Rudolph J. Schmid R. Höper D. Engelmann S.. (2001). Extracellular proteins of *Staphylococcus aureus* and the role of SarA and sigma B. Proteomics 1, 480–493. 10.1002/1615-9861(200104)1:4<480::AID-PROT480>3.0.CO;2-O 11681202

